# KomoTrip: a multi-day travel itinerary recommendation method based on the discrete komodo mlipir algorithm

**DOI:** 10.7717/peerj-cs.3350

**Published:** 2025-11-12

**Authors:** Z. K. Abdurahman Baizal, Soni Fajar Surya Gumilang, Rio Nurtantyana, Rahmat Hendrawan

**Affiliations:** 1 School of Computing, Telkom University, Bandung, West Java, Indonesia; 2 School of Industrial Engineering, Telkom University, Bandung, West Java, Indonesia; 3 National Research and Innovation Agency, Bandung, Indonesia; 4 Telkom Indonesia, Jakarta, Indonesia

**Keywords:** Multi-day routes recommender system, Weighted multi-attribute user preference, Team orienteering problem with time windows, Multi-attribute utility theory

## Abstract

Technological developments in recent years led to the emergence of increasingly sophisticated recommender systems to support multi-day travel itineraries that fall under the Tourist Trip Design Problem (TTDP). Various problem analogies are widely used to solve TTDP, such as Traveling Salesman Problem (TSP), Vehicle Routing Problem (VRP), Orienteering Problem (OP), and Team Orienteering Problem with Time Windows (TOPTW). For multi-day route recommendation, TOPTW is suitable as a problem analogy since there is a per-day travel duration constraint. So far, TTDP with TOPTW does not consider the weighting (priority level of users) for each requirement attribute in a multi-attribute-based TOPTW to ensure personalized recommendations. In addition, running time remains a challenge in many studies in the TOPTW area. Many metaheuristic algorithms have been adopted to TOPTW for generating a time-efficient approach. Komodo Mlipir Algorithm (KMA) emerges as a new algorithm that promises good scalability. Therefore, we propose KomoTrip, a method that adopts the discrete version of KMA and Multi-Attribute Utility Theory (MAUT) to recommend optimal travel routes per day by accommodating the multi-attribute preferences of users. We perform three evaluation scenarios, *i.e*., general performance, Degree of Interest (DOI) combinations, and varying numbers of Points of Interest (POI), consistently demonstrating that KomoTrip outperforms several benchmark algorithms in terms of computational time efficiency and also exhibits robust fitness values across different problem dimension scales. Thus, KomoTrip can be regarded as an efficient algorithm to recommend optimal multi-day tour routes, effectively incorporating weighted multi-attribute preferences into its optimization process. We further benchmarked KomoTrip against state-of-the-art TOPTW heuristics on the public Solomon dataset, where it demonstrated competitive profit values, particularly for a larger number of days (tours), and consistently achieved superior runtime performance.

## Introduction

Tourism becomes an important part of the urban lifestyle. Vacations are now used not only to rest, but also to explore and enjoy new experiences in various tourist destinations. The development of technology plays a major role in making it easier for people to access tourist information through applications such as TripAdvisor, Google Maps, and Traveloka, which provide references to tourist attractions based on user reviews. In addition, research focusing on user-driven travel recommender systems also grows rapidly, providing alternatives for travelers to get travel experiences that match their preferences (Abbasi-Moud et al., 2022; Wang, 2023; Fudholi et al., 2023). Through the increasing number of destination options and the ease of information available, travelers are encouraged to design more compact and diverse trips. Most travelers want to visit several tourist destinations in one day. Therefore, their needs are not only limited to recommendations for tourist attractions, but also include optimal travel routes.

There are many efforts to fulfill the need for optimal travel routes, especially through the development of recommender systems for 1-day tours (Sylejmani, Dorn & Musliu, 2017; Ji et al., 2021; Wu et al., 2022; Wang & Hu, 2023). However, the focus on 1-day trips becomes a limitation, as many travelers now tend to prefer multi-day trips to explore a region in more depth. This condition drives the need for a recommender system that not only recommends destinations, but is also able to generate optimal multi-day travel itineraries. Some studies try to address this need by adapting the multi-day travel routing problem to the Traveling Salesman Problem (TSP) analogy (Fahlevi & Baizal, 2023; Mahardika & Baizal, 2023). The goal of TSP is to find a single optimal route visiting all cities once and returning to the origin (Hao et al., 2023; Ergüven & Polat, 2024). While this suits 1-day tours, applying it to multi-day trips (by dividing the full route into daily segments) often leads to suboptimal daily routes due to the lack of per-day time considerations. However, since the TSP is conceptually optimized for one whole trip without considering the number of visit days and daily trip duration, this truncation process results in subroutes that are not optimal as per-day trips. In other words, the TSP analogy is more relevant for single route optimization, such as in a 1-day tour.

To address TSP limitations in multi-day trip planning, recent studies analogizes it as a Vehicle Routing Problem (VRP), where each vehicle represents a day’s route using Brain Storm Optimization (BSO) (Hendrawan, Baizal & Wulandari, 2024), Improved Whale Optimization Algorithm with Variable Neighborhood Search Strategy (WOA-VNS) (Ananda, Baizal & Wulandari, 2024), Hybrid Genetic Algoritm and Simulated Annealing (GA-SA) (Istiqfarri, Baizal & Wulandari, 2025). This approach supports constraints, such as daily duration and attraction hours, using variants such as Capacitated VRP with Time Windows (CVRPTW) and VRP with Time Windows (VRPTW). In CVRPTW, visit duration is treated as vehicle capacity, time windows reflect attraction hours, and unvisited Points of Interest (POI) receive penalties. Two other studies use the VRPTW approach, by integrating visit duration and destination operating hours constraints as vehicle and POI time windows, and dividing routes with a greedy strategy that considers all POIs (Ananda, Baizal & Wulandari, 2024; Istiqfarri, Baizal & Wulandari, 2025). Although this VRP-based approach produces optimal multi-day routes compared to the TSP approach, there is a fundamental limitation because the classic VRP remains oriented towards servicing routes that seek as many visits as possible, not on selecting the best subset of destinations. In travel planning, travelers do not need to, and often cannot, visit all available destinations, but rather select only those that are most relevant to their preferences within the constraints of time and daily visit duration. Therefore, a more suitable approach to describe such needs is the Team Orienteering Problem (TOP).

TOP is an extension of the Orienteering Problem (OP) and also includes a variant of Profit VRP (PVRP) (Vansteenwegen & Gunawan, 2019; Chaigneau, Bostel & Grimault, 2025). TOP is an Nondeterministic Polynomial time (NP)-hard combinatorial problem, so its solution demands efficient optimization approaches to handle the large number of possible solutions (Mısır, Gunawan & Vansteenwegen, 2022). In general, TOP aims to select a subset of available points to visit by maximizing the total profit earned without exceeding the predefined travel time limit for each route (Chao, Golden & Wasil, 1996). In its application to the multi-day travel route problem, TOP is used to select the most valuable destinations for tourists, taking into account the travel time constraints of each day. Each generated route represents 1 day of travel, thus allowing for more realistic visit planning that matches the user’s preferences. However, in the classic TOP model, the visit time to each destination is considered flexible as long as it does not exceed the trip duration limit, without considering the opening and closing hours of each destination. Meanwhile, in real-life travel planning, each POI has certain operating hours that limit when the destination can be visited. To accommodate this need, the Team Orienteering Problem (TOP) is extended into the Team Orienteering Problem with Time Windows (TOPTW). In this variant, each destination must be visited within a specified time window while still respecting the overall trip duration limit (Amarouche et al., 2020).

In the TOPTW-based multi-day travel route problem, previous studies only optimize profit and accessibility (El-Hajj et al., 2015; Hu, Fathi & Pardalos, 2018; Gavalas et al., 2019), or the total score of POIs visited (Vansteenwegen et al., 2009; Gunawan, Lau & Lu, 2016), without considering the priority of user preferences, which should be an important factor in the preparation of personalized itineraries. Since the Tourist Trip Design Problem (TTDP), or in this case, we call it multi-day routes, is an NP-hard problem, execution time remains a challenge that is not fully resolved, although many approaches produce relatively optimal solutions (in terms of fitness value). Many metaheuristic algorithms are used to solve TOPTW in Tourist Trip Design Problem (TTDP), such as Simulated Annealing (SA) (Tlili & Krichen, 2021), Ant Colony Optimization (ACO) and Ant Colony System (ACS) (Verbeeck, Vansteenwegen & Aghezzaf, 2017), Tabu Search (TS) (Sylejmani, Dorn & Musliu, 2012), Multi-Objective Genetic Algorithm (MOGA) (Moosavi Heris et al., 2022), Discrete Particle Swarm Optimization (DPSO) (Yu et al., 2019), and a three-step TTDP approach integrating Modeling to Generate Alternatives (MGA) and contextual factors (Pérez-Cañedo et al., 2024). In addition, a recent study introduced a local search method for an extended TTDP variant with POI category sequencing, achieving solutions comparable to state-of-the-art Iterated Local Search (ILS)-based solvers on 146 benchmark instances (Sylejmani et al., 2024). Therefore, an optimization method is needed that can produce recommendations in a short time, while maintaining optimality. Komodo Mlipir Algorithm (KMA) is a new optimization algorithm that is able to guarantee the achievement of globally optimal solutions with a very good level of scalability (Suyanto, Ariyanto & Ariyanto, 2022; Jati et al., 2023).

This research proposes KomoTrip, a method for solving multi-attribute TOPTW in multi-day trip planning. It allows users to assign explicit weights to each attribute (criterion) based on their preferences. KomoTrip uses a modified Discrete Komodo Algorithm in recommending optimal routes. In recommender systems, this approach belongs to constraint-based recommender systems. In this study, we employ a proprietary dataset of Yogyakarta tourist destinations, Indonesia, to develop our KomoTrip. For evaluation and benchmarking, we additionally incorporate public instances from the Solomon dataset. In summary, the main contributions of this research consist of:

1. We proposes a multi-attribute-based routes recommendation approach that considers the priority level (weight) of the user’s interest in each attribute (criteria) requirement in the TOPTW problem analogy. These preference weights are expressed through Degree of Interest (DOI) for each requirement attribute.
2. We propose the KomoTrip method, which is an adaptation of the Discrete Komodo Algorithm (DKA) to solve TOPTW, specifically to generate personalized multi-day routes recommendation based on multi-attribute user preferences, consists of:
  1) Algorithm for multi-day route split,
  2) Customized DKA for TOPTW solving, and
  3) Optimization model for personalized multi-day routes recommendation. This model includes defining the objective function, constraint function, penalty function, and also utilizing Multi-Attribute Utility Theory (MAUT) to accommodate multi-attribute user preferences.

The remainder of this article is organized as follows. Section ‘Related Work’ discusses foundational and recent developments in travel route planning, particularly in the context of combinatorial optimization problems such as the Traveling Salesman Problem (TSP), Vehicle Routing Problem (VRP), Orienteering Problem (OP), and Team Orienteering Problem with Time Windows (TOPTW). The next part, Section ‘Classic Komodo Mlipir Algorithm (KMA)’, describes the original algorithm of KomoTrip, the Classic KMA, providing background on its mechanisms and limitations. In Section ‘Optimization Modeling in KomoTrip’, we elaborate on the optimization model used in KomoTrip, focusing on the constraints and objective function. This is followed by Section ‘Algorithm for Finding the Optimal Solution in KomoTrip’, which outlines the method we develop to generate optimal solutions. Section “Experimental Results” presents the experimental results from various tested scenarios. Finally, the ‘Conclusion’ section summarizes this study by highlighting the performance of KomoTrip and suggesting directions for future research.

## Related work

The Orienteering Problem (OP) is first introduced by Tsiligirides (1984), who formulates the challenge in the sport of orienteering as a combinatorial optimization problem, in which participants must select a subset of visit points to obtain the highest possible score within a given time constraint. This problem shares a structural similarity with the Traveling Salesman Problem (TSP), as both involve planning a visit route, but differ in objectives and constraints: TSP requires visiting all points to minimize distance, while OP selects only some points to maximize score (Vansteenwegen, Souffriau & Oudheusden, 2011). As real-world needs evolve, especially in cases involving more than one agent or vehicle, OP is later extended into the Team Orienteering Problem (TOP). Chao, Golden & Wasil (1996) develops the basic formulation of TOP and proposes a heuristic approach to solve multiple visit routes simultaneously. Since then, TOP continues to be the focus of many studies across various fields such as logistics distribution and travel planning, giving rise to numerous model variants tailored to the needs of each domain.

Among the developed variants, two that are most frequently discussed in the literature are the Orienteering Problem with Time Windows (OPTW) and the Team Orienteering Problem with Time Windows (TOPTW). Vansteenwegen et al. (2009) states that TOPTW is one of the most relevant variants for real-world cases such as tour planning, as it considers the operating time of each visit location. In another study, Gunawan, Lau & Vansteenwegen (2016) also emphasizes that OPTW and TOPTW are central to the development of realistic route planning models, particularly in the logistics and tourism domains. One study that adopts OPTW in the Tourist Trip Design Problem (TTDP) is Abbaspour & Samadzadegan (2011), which develops a tourist route model in urban areas by taking into account travel time and transportation conditions. Meanwhile, Gavalas et al. (2013) formulates TTDP as a TOPTW and proposes two cluster-based heuristic algorithms, CSCRatio and CSCRoutes, to generate efficient multi-day itineraries by considering profit, visit time, and the spatial distribution of POIs. Although these approaches improve solution quality compared to the standard Iterated Local Search (ILS), such heuristic methods still have limitations in global solution exploration.

To overcome these limitations, many recent studies begin utilizing metaheuristic algorithms to explore the solution space more extensively and to find higher-quality routes for solving TOPTW. For example, Tlili & Krichen (2021) proposes a hybrid approach that combines K-Means clustering to divide multi-day routes and Simulated Annealing (SA) to optimize per-day routes, aiming to maximize the total score of visited POIs. Similarly, Sylejmani, Dorn & Musliu (2012) develops the Multi-Constrained Team Orienteering Problem with Time Windows (MCTOPTW) model and applies the Tabu Search (TS) algorithm to find optimal route solutions. Khodadadian et al. (2022) explores the Ant Colony System (ACS) in the context of the Time-Dependent Orienteering Problem with Time Windows (TD-OPTW). In addition, Yu et al. (2019) introduces a variant called the Team Orienteering Problem with Time Windows and Partial Scores (TOPTW-PS), and proposes the Selective-Discrete Particle Swarm Optimization (S-DPSO) algorithm as the solution method. Another development is carried out by Moosavi Heris et al. (2022), who propose a different approach using a Multi-Objective Genetic Algorithm (MOGA) to optimize routes in TOPTW by considering several constraints such as profit maximization and accessibility. A recent study introduced a three-step TTDP methodology using MGA and fuzzy propositions to incorporate contextual factors and traveler needs (Pérez-Cañedo et al., 2024). More recently, a local search method has been proposed for an extended TTDP variant with POI category sequencing, which achieved solutions comparable to state-of-the-art ILS-based solvers on 146 benchmark instances (Sylejmani et al., 2024).

Although these studies have successfully improved the effectiveness of TOPTW solutions, most still focus on maximizing score, profit, or other general objectives without explicitly incorporating preference weighting across user attributes (for example, relying only on a single dimension of priority). In addition, execution time remains a key issue in generating optimal travel route recommendations (Gunawan, Lau & Vansteenwegen, 2016). One of the relatively recent metaheuristic algorithms designed to maintain a balance between the exploration and exploitation phases is the Komodo Mlipir Algorithm (KMA) (Suyanto, Ariyanto & Ariyanto, 2022). To efficiently solve the discrete nature of multi-day route recommendation, this study adopts the Discrete Komodo Algorithm (DKA), which balances solution quality and computation time. DKA uses edge-construction and edge-destruction for exploitation, and insertion and 2-opt swap for exploration, to effectively navigate the discrete solution space (Jati et al., 2023). Building on this, we propose KomoTrip, an adaptation of DKA for the TOPTW problem, that incorporates Degree of Interest (DOI) using Multi-Attribute Utility Theory (MAUT) to align recommendations with user preferences. Additionally, a greedy-based split_itinerary algorithm (Algorithm 1) is introduced to segment the overall route into per-day trips and integrate it within the optimization model.

**Algorithm 1:** Itinerary separation.

1: **Function** split_itinerary*(P)*
2: {*P* is the existing single-route itinerary}
3: $P^{\prime} \leftarrow \left[ \, \right]$ {initiate empty itinerary}
4: day ← 1
5: ${P_{unavailable}} \leftarrow \left[ \, \right]$ {initiate empty list of unavailable POIs}
6: **while** day $\le n$ **do**
7: position $\leftarrow {p_{0}}$ {set initial position to hotel}
8: ${D_{day}} \leftarrow \left[ \, \right]$ {initiate empty list for this day itinerary}
9: ${P_{next}} \leftarrow$ All POIs in *P* but not in *P*_*unavailable*_ {a list of next POI candidates}
10: **for all** *i* in *P*_*next*_ **do**
11: **if** *p*_*i*_ is available to visit (Eqs. (13), (15) and (16)) **then**
12: add *p*_*i*_ to *D*_*day*_ and *P*_*unavailable*_
13: position $\leftarrow {p_{i}}$
14: **end if**
15: **end for**
16: **if** *D*_*day*_ is not empty **then**
17: add *D*_*day*_ to *P’*
18: **end if**
19: **if all** *P* in *P*_*unavailable*_ **then**
20: break the iteration
21: **end if**
22: day ← day+1
23: **end while**
24: **return** *P′*

## Classic komodo mlipir algorithm (KMA)

Komodo Mlipir Algorithm (KMA) is one of the swarm intelligence algorithms inspired by the behavior of Komodo (Suyanto, Ariyanto & Ariyanto, 2022). KMA works by dividing the population into three types of Komodo, *i.e*., big males, females, and small males. The mapping of each individual (Komodo) into the three types is done based on the quality of each individual, where big males represent high-quality individuals, females represent medium-quality individuals, and small males represent low-quality individuals. Each type of Komodo has a different search strategy (movement) as follows:

1. Big males have two movements: attraction and distraction. Attraction occurs when a big male approaches another bigger male or vice versa, while distraction occurs when a big male moves away from another smaller male. The focus of the big males’ movement is High Exploitation Low Exploration (HILE). HILE is indicated by more big Komodo moving closer to the bigger male (better solution) than away from the smaller male (worse solution), so that exploitation of the solution is favored in the movement of the big male.
2. Females have two movements, *i.e*., mating and parthenogenesis. Mating occurs when a female mates with the largest male, while parthenogenesis occurs when a female performs asexual reproduction. The focus of the female’s movement is Medium Exploitation Medium Exploration (MIME). MIME is indicated by an equal weight between movement towards the larger male and random movement (parthenogenesis).
3. Small males have one movement, which is the mlipir movement. Mlipir is a movement that approaches the big male indirectly. The focus of small males’ movement is Low Exploitation High Exploration (LIHE). LIHE is shown by more small males performing mlipir movement, which involves approaching the big male (better solution) indirectly (turning through the distance), so the solution exploration process is prioritized in the small male movement.

KMA includes a parameter $p$ in the range of $(0,1)$, which serves to split the population into three types of komodos. Based on the parameter $p$, the population of $n$ komodos is divided into $q$ big males, one female, and $s$ small males, as in Eqs. (1) and (2).

(1) $$q = \lfloor (p - 1)n\rfloor$$

(2) $$s = n-q.$$

The movement of big males ${k_{i}}$ results in a new position of $k^{\prime} i$, which is defined as in Eq. (3), where $f({k_{i}})$ and $f({k_{j}})$ are the qualities of the komodos ${k_{i}}$ and ${k_{j}}$. The quality of the komodos is indicated by the result of the fitness calculation on the solution represented by the komodos. The change in position of Komodo ${k_{i}}$ with respect to Komodo ${k_{j}}$ is indicated by ${w_{ij}}$. In addition, ${r_{1}}$ and ${r_{2}}$ are random numbers in the range $[0,1]$.

(3) $$k^{\prime} _{i} = {k_{i}} + \sum\nolimits_{j = 1}^q {{w_{ij}}} ,\;\rm {where} \;j \ne i$$

(4) $${w_{ij}} = \left\{ {\matrix{ {{r_{1}}({k_{j}} - {k_{i}}),} \hfill & {f({k_{j}})< f\left( {{k_{i}}} \right)\;\rm {or} \;{r_{2}}\lt0.5} \hfill \cr {{r_{1}}({k_{i}} - {k_{j}}),} \hfill & {otherwise} \hfill \cr } } \right..$$

Female movement is determined with a probability parameter of 0.5. Females can exploit by mating with the largest big male and produce two new generations, as defined in Eq. (5). The symbols ${k_{il}}$ and ${k_{jl}}$ represent the largest big male and female komodo, while $k^{\prime} _{il}$ and $k^{\prime} _{jl}$ are the two new generations resulting from the mating. Furthermore, the female Komodo will be replaced by the best new generation.

(5) $$\eqalign{k^{\prime} _{il} & = {r_{l}} \cdot {k_{il}} + (1 - {r_{l}}) \cdot {k_{jl}}\\ k^{\prime} _{jl} & = {r_{l}} \cdot {k_{jl}} + (1 - {r_{l}}) \cdot {k_{il}}}.$$

The second movement of the female Komodo is parthenogenesis, where the female Komodo will explore by expanding the search area. Parthenogenesis is defined in Eq. (6) where ${k_{ij}}$ is the $j$-th value of the Komodo ${k_{i}}$, $j \in \{ 1,2,3, \ldots ,m\}$, and ${k_{ij}} \in [l{b_{j}},u{b_{j}}]$. The symbols $l{b_{j}}$ and $u{b_{j}}$ are the lower bound and upper bound of komodo ${k_{i}}$, $r$ is a random number $[0,1]$, and $\alpha$ is the parthenogenesis radius of 0.1.

(6) $$k^{\prime} _{ij} = {k_{ij}} + \left( {2r - 1} \right)\alpha |u{b_{j}} - l{b_{j}}|.$$

Small males perform mlipir movements that are more exploration than exploitation. The mlipir is performed by randomly selecting the dimension of the small male komodo based on the mlipir rate $d$, which has an interval of $(0,1)$. The mlipir movement is defined in Eq. (7).

(7) $$k^{\prime} _{i} = {k_{i}} + \sum\limits_{j = 1}^q {{w_{ij}}} ,\; {\rm where} \;j \ne i$$

(8) $${w_{ij}} = \left\{ {\matrix{ {\sum\nolimits_{l = 1}^m {{r_{1}}} ({k_{jl}} - {k_{il}}),} \hfill & {{r_{2}}< d} \hfill \cr {0,} \hfill & {otherwise} \hfill \cr } } \right..$$

The notations ${r_{1}}$ and ${r_{2}}$ are random numbers in the range $[0,1]$, where ${r_{1}}$ indicates the mlipir movement speed and ${r_{2}}$ selects the dimension followed. The notations ${k_{il}}$ and ${k_{jl}}$ represent the $l$-th dimension of the small male and big male, where $l$ is a randomly selected dimension with the same probability as the mlipir rate. In addition, the number of Komodo big males is represented by $q$.

KMA consists of two phases that enable it to cope with unimodal and multimodal functions effectively and efficiently. The first phase is conducted for 1,000 generations (1,000 iterations) with a small population size, and $p = 0.5$. Furthermore, the second phase is performed with a large and adaptive population size. This causes KMA to potentially have a higher running time because it has to search for solutions twice.

The adaptive population size in phase 2 is regulated by an adaptation scheme. If the last two best fitness values increase, then the population size $m$ will be reduced by a number of komodos. Conversely, if the last two best fitness values do not increase, the population size will be increased by a new number of komodos. The new population size $m^{\prime}$ can be defined as in Eq. (9), where ${f_{1}},{f_{2}},{{\mathrm{and}}}\ {f_{3}}$ are the $i$-th best fitness values.

(9) $$m^{\prime} = \left\{ {\matrix{ {m-a,} \hfill & {\delta {f_{1}} > 0\; {\rm and} \;\delta {f_{2}} > 0} \hfill \cr {m + a,} \hfill & {\delta {f_{1}} = 0\;\rm {and} \;\delta {f_{2}} = 0} \hfill \cr } } \right.$$

(10) $$\delta {f_{1}} = {{|{f_{1}} - {f_{2}}|} \over {{f_{1}}}}$$

(11) $$\delta {f_{2}} = {{|{f_{2}} - {f_{3}}|} \over {{f_{2}}}}.$$

Our study performs optimization on tourist routes that are analogous to TOPTW. Therefore, it can be said that the optimization carried out is discrete. The optimization process in KMA can only be applied to continuous problems, so adjustments need to be made for discrete problems. In TOPTW, optimization is done to find the most optimal sequence of nodes. If *N* is the set of nodes to be optimized, then one Komodo is a vector with length |*N*| that has an initial value in the range $[0,1]$ (during the process, the value can go out of this range). Each element in the Komodo vector represents a node, and the value of each element represents the order of the nodes in the resulting route. For example, for $N = \{ 0.2,0.3,1,0.1\}$, the resulting route is ${N_{3}},{N_{2}},{N_{1}},{N_{4}}$ (sorted in descending order).

## Optimization modeling in KomoTrip

KomoTrip recommends multi-day routes based on multi-criteria user preferences. User preferences are expressed directly by the user, just like in a knowledge-based recommender system. We utilize the constraint-based recommender system approach, in the knowledge-based recommender system paradigm. Users express their preferences explicitly through one shot interaction in a conversational recommender system.

The recommendation of tourist routes in this study is formulated as an optimization problem involving user-side preferences with three criteria, representing the common preferences of a tourist. However, this model is also applicable to more criteria because it is built generically. The three criteria are as follows:

1) As many POIs as possible are included in the itinerary
  This requirement is expressed by the user, but in modeling, we translate it as a function that minimizes travel duration. By minimizing travel duration, more tourist destinations will be visited. However, we still need to balance two functions: maximizing the number of POIs in the itinerary and minimizing travel duration. In this case, users focus solely on the number of tourist destinations visited without being overly concerned with the popularity of the destination (POI) or the budget.
2) Itinerary consisting of popular POIs
  Users may want to visit popular tourist destinations. The popularity value of a POI is seen from the rating obtained. This rating data is obtained from the Google Serp Application Programming Interface (API). In terms of modeling, we can consider this as a function that maximizes the average rating of each POI. However, if we only focus on this function, the itinerary may only consist of one POI. Therefore, there are two functions that must be considered: maximizing the average rating of each POI visited and also maximizing the number of POIs included in the itinerary.
3) Budget-minimizing itinerary
  Not all tourist attractions are affordable for certain tourists, so many tourists consider the total budget in their tour. In modeling, we can represent this need as a function that minimizes the total entry cost. This study does not consider parking fees, as parking fees in Indonesia are generally the same. However, in the optimization model, to achieve this goal, two functions must be considered simultaneously: minimizing the budget and maximizing the number of POIs in the itinerary. If we only consider the budget minimization function, the itinerary may consist of only one POI.

The fulfillment of these three preferences becomes the objective function of the optimization problem. If we look back, each of these user preference criteria should result in as many POIs in the itinerary as possible. This preference consideration is also included in the objective function, which will be discussed in Section ‘Modeling for the Objective Function’. If these conditions are not present, then the recommended route will not reflect the user’s preferences. In this study, we define user preferences, constraint function, and objective function in the TOPTW optimization problem. In addition, we define the penalty function as a part of the objective function.

## Dataset

In this research, we use a dataset of tourist destinations in the Yogyakarta region of Indonesia (https://doi.org/10.5281/zenodo.17053243). Yogyakarta is the second most popular tourist destination in Indonesia, following Bali. The dataset includes POI name, geographic coordinates (latitude and longitude), opening and closing hours, average time spent by people at one POI, rating of each POI, and entrance fee, which we get from the Google Serp API. In addition, we use the Google Maps API to get travel duration data between POIs. The POIs data taken consists of 87 destinations, which include several categories such as nature tourism, religion, history, education, shopping, games, and culture and heritage. For benchmarking, we additionally incorporate public instances from the Solomon dataset (https://www.mech.kuleuven.be/en/cib/op).

### Modeling for constraint function

Before we discuss the objective function, we first examine the constraint function in the optimization problem. In this research, we consider the optimization problem as a solution to TOPTW. Thus, the constraint function in TOPTW, as it relates to the tourist route recommendation case, can be considered the constraint function of the optimization problem.

**Figure 1:**
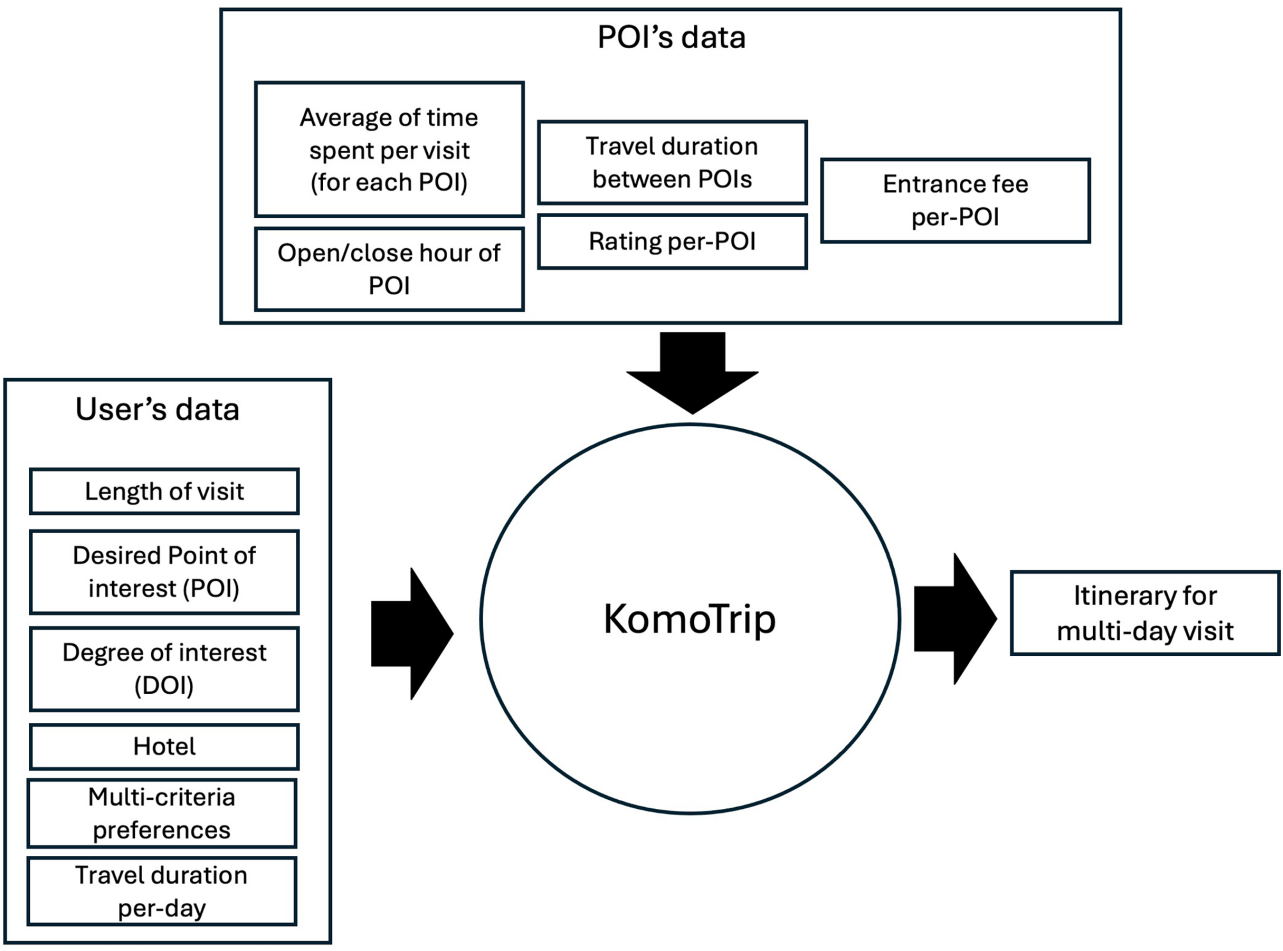
Data processed in KomoTrip.

**Figure 2:**
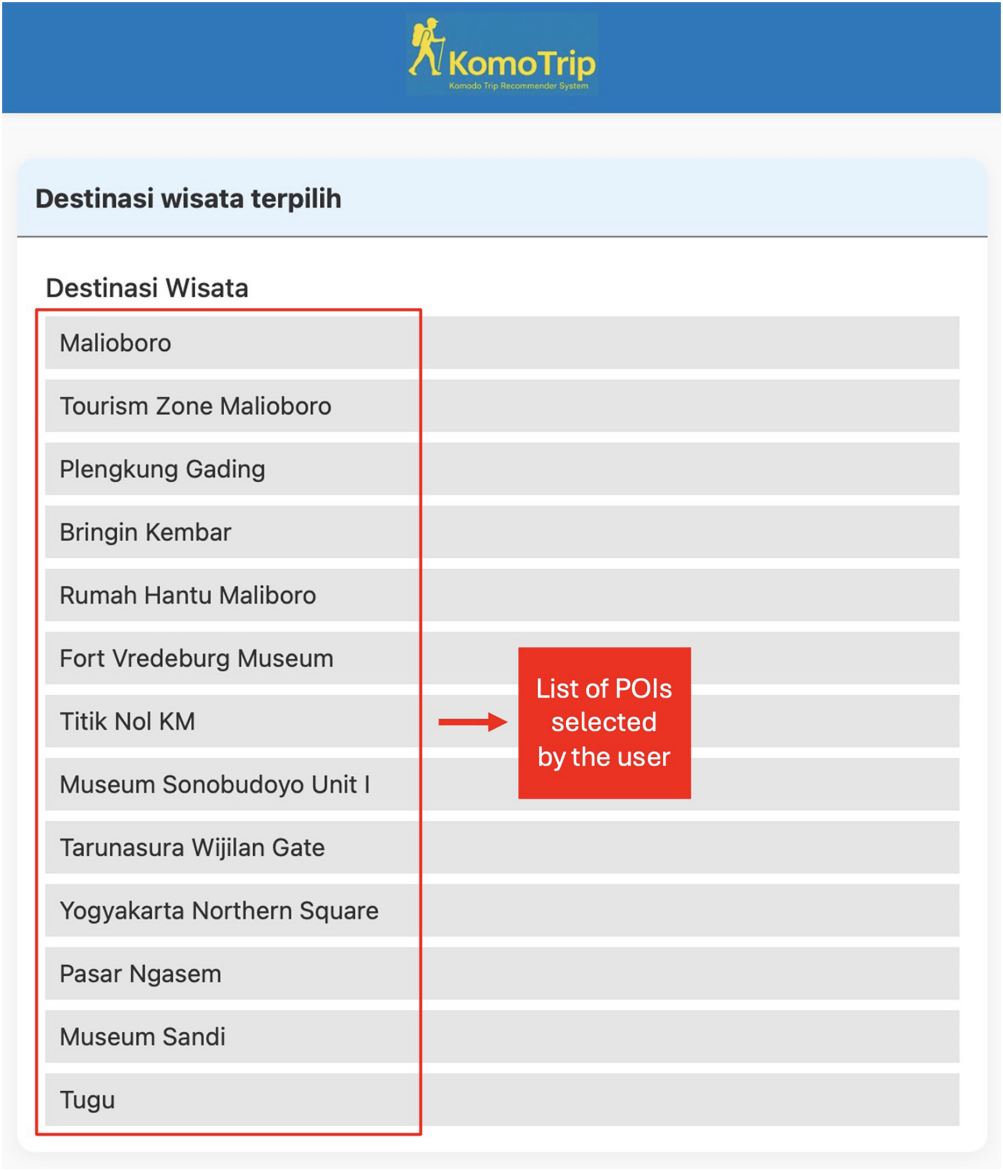
Screenshot of the user interface showing the list of POIs selected by the user.

Figure 1 shows the data to be processed in KomoTrip, while Fig. 2 shows the list of POIs selected by the user in our application (KomoTrip (https://go-routes.com)). The personalized itinerary recommendation generated by KomoTrip is based on user input, which includes hotel selection and desired POIs. We denote the set of hotels and POIs desired by the user as a set. Tourists are also allowed to specify the Degree of Interest (DOI) for each requirement criterion (attribute). The DOI represents the tourist’s interest weight for each requirement criterion, where ${{\mathrm{DOI}}} \in [0,1]$. Additionally, the tourist (user) can define the number of days they will spend on vacation $(n)$. The system’s task is to recommend an optimal tourist route in the form of a travel itinerary consisting of a set of POIs to be visited over several days. For example, if the set of POIs in the itinerary is denoted as *P*, and the set of days in the itinerary (day 1, day 2, $\ldots$) is denoted as *D*, then we can define,

(12) $$\eqalign{|P| & \le |\rho |\\|D| & \le n}.$$

Let *D* be the set of days, with ${d_{i}} \in D$ representing the $i$-th day, where $i = 1,2,3, \ldots ,|D|$. Meanwhile, *P* is the set of POIs included in the itinerary and ${p_{i}}$ is the $i$-th POI visited, $i = 0,1,2,3, \ldots ,|P|$, where ${p_{0}}$ represents the hotel. The route for each ${d_{i}}$ consists of traveling from ${p_{0}}$ (hotel), visiting each ${p_{i}}$ exactly once, and returning to ${p_{0}}$. To ensure that each ${p_{i}}$ is visited only once in the route (except ${p_{0}}$), the generated route must satisfy the following equation,

(13) $$\eqalign{&\sum_{d_i \in D} \sum_{p_i \in P} x_{p_i p_j d_i} = 1, \quad \forall p_j \in P, \; (i \neq j)  \\&  \sum_{d_i \in D} \sum_{p_j \in P} x_{p_i p_j d_i} = 1,  \quad \forall p_i \in P, \; (i \neq j) .}$$

The notation ${x_{{p_{i}}{p_{j}}{d_{i}}}}$ represents the presence or absence of trips from ${p_{i}}$ to ${p_{j}}$ on day ${d_{i}}$, as defined by the following formula,

(14) $${x_{{p_{i}}{p_{j}}{d_{i}}}} = \left\{ {\matrix{ {1,} \hfill & {{\mathrm{if\;there\;is\;a\;trip\;from\;}} {p_{i}}{\mathrm{\;to\;}} {p_{j}}{\mathrm{\;on\;day\;}} {d_{i}}} \hfill \cr {0,} \hfill & {otherwise} \hfill \cr } } \right..$$

The travel duration per day in the generated route should not exceed the maximum travel duration per day. For example, if the tourist sets to depart from the hotel at 8:00 AM and must return no later than 8:00 PM, then the maximum travel duration is 12 h. Henceforth, the notation $\tau$ represents the maximum limit of travel duration, which can be set by the tourist. Each POI has an opening hour ${o_{{p_{i}}}}$ and a closing hour ${c_{{p_{i}}}}$. If the tourist arrives at ${p_{i}}$ before ${o_{{p_{i}}}}$, then he/she has to wait. Therefore, we define a waiting time variable as $t{w_{{p_{i}}}}$. Besides waiting time, the variable we need to consider is the time spent by a tourist at ${p_{i}}$, denoted as $t{s_{{p_{i}}}}$. We obtain the average time spent by tourists at ${p_{i}}$ from Google and use this data to assign the value of $t{s_{{p_{i}}}}$. The time taken to travel from POI ${p_{i}}$ to ${p_{j}}$ is denoted as ${t_{{p_{i}}{p_{j}}}}$. The value of ${t_{{p_{i}}{p_{j}}}}$ is obtained from the Google Serp API. Since the per day travel route cannot exceed the maximum daily travel limit ( $\tau$), we can define a capacity constraint in TOPTW as follows,

(15) $$\eqalign{&t_{tot} = \sum_{p_i \in P} \sum_{p_j \in P}  \left( t_{p_i p_j} + tw_{p_j} + ts_{p_j} \right) \cdot x_{p_i p_j d_i} \le \tau, \forall d_i \in D, \;(j \ne 0)\\& t{w_{pi}} = t{s_{{p_{i}}}} = 0,\;{\rm {for\;}} {p_{0}}{\mathrm{\;(hotel)}.}}$$

In a tourist visit, tourists must complete the visit at ${p_{i}}$ before ${c_{pi}}$. We define this condition as a time window constraint in TOPTW, by using the following formula,

(16) $${o_{{p_{i}}}} \le a{t_{{p_{i}}}} + t{w_{{p_{i}}}} + t{s_{{p_{i}}}} \le {c_{pi}},\;\;\forall {p_{i}} \in P,\;\;i \ne 0$$

where $a{t_{{p_{i}}}}$ is the arrival time of the tourist at ${p_{i}}$.

**Figure 3:**
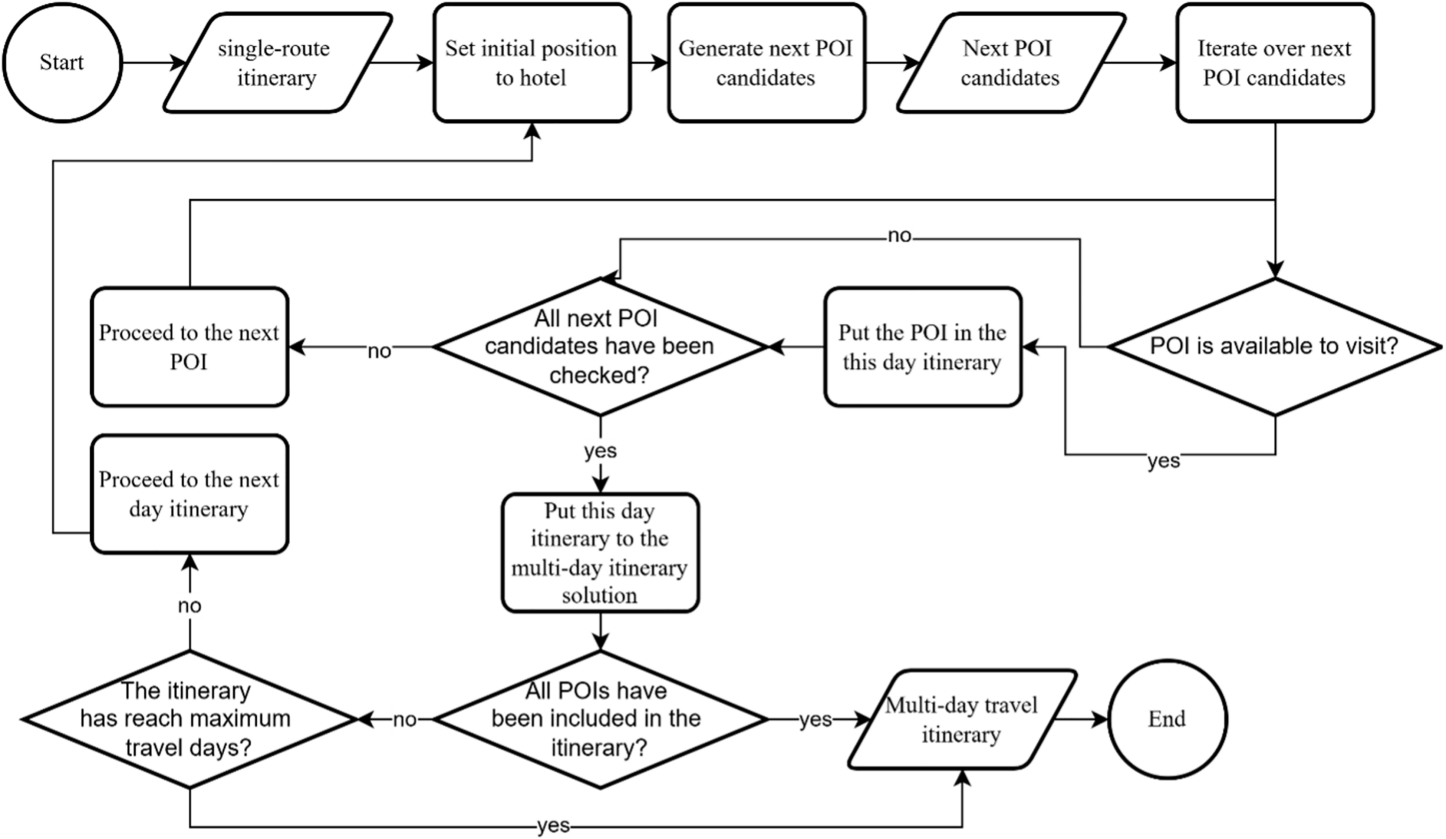
Flowchart split itinerary.

The solution generated in KomoTrip must fulfill the constraints defined as shown in Fig. 3 (the split_itinerary flowchart). The splitting process is performed by checking each POI in the itinerary and placing it in the daily itinerary in the multi-day travel itinerary. Each POI will be checked to see whether it can still be visited or not until all POIs are included in the itinerary or the itinerary has reached the maximum number of tourist days. A more detailed description is contained in Algorithm 1, which is an algorithm that serves to break the itinerary into multi-day itineraries that satisfy all the constraints. Algorithm 1 starts by accepting a single-route itinerary as input. On line 5, there is a list stores POIs that have already been visited. This is useful for fulfilling the constraints in Eq. (13). Lines 6 to 22 outline the steps for splitting the itinerary according to the maximum number of days to fulfill the constraints in Eq. (12). The next step is to check the closing hours at the next candidate POI to ensure the resulting solution fulfills the constraint function in Eq. (16) (line 11). In addition, line 13 checks whether the next candidate POI can still be visited based on the constraints in Eqs. (12) and (15). Finally, Algorithm 1 produces a new solution in the form of an optimal multi-day itinerary that satisfies all existing constraints.

### Modeling for the objective function

As discussed in the previous sections, user preferences can be considered as three criteria (attributes) that should be considered: (1) minimize the travel duration, (2) maximize the average rating of each POI visited, and (3) minimize the total budget. These three criteria become part of the objective function of the optimization problem in TOPTW. Referring to these criteria, we can again write down three attributes in the formulation of the objective function:

(1) Total travel duration $({t_{{{\mathrm{tot}}}}})$,
(2) Average rating $(\bar r)$,
(3) Entrance fee $(f)$.

We formulate the total travel duration over |*D*| days of visit $({t_{{{\mathrm{tot}}}}})$ as follows,

(17) $${t_{tot}} = \sum\limits_{{d_{i}} \in D} {\sum\limits_{{p_{i}} \in P} {\sum\limits_{{p_{j}} \in P} {\left( {{t_{{p_{i}}{p_{j}}}} + t{w_{{p_{j}}}} + t{s_{{p_{j}}}}} \right)} } } \cdot {x_{{p_{i}}{p_{j}}{d_{i}}}}\quad j \ne 0.$$

Meanwhile, we define the average attribute rating $(\bar r)$ as,

(18) $$\bar r = {{\sum\nolimits_{{p_{i}} \in P}^{|P|} {{r_{{p_{i}}}}} } \over {|P-\left\{ {{p_{0}}} \right\}|}}\quad i \ne 0.$$

and the entrance fee attribute $(f)$ as,

(19) $$f = \sum\limits_{{p_{i}} \in P} {{f_{{p_{i}}\quad}}} i \ne 0.$$

In this study, we define two penalty functions to help find the optimal solution, *i.e*.,:

1. Penalty related to the number of desired POIs that are not included in the itinerary (recommended route),
(20) $$pe{n_{POI}} = |\rho - P|.$$
2. Penalty related to the excess travel duration per day,
(21) $$pe{n_{time}} = \max \{ ({T_{d}} - \tau ),0\},$$

where ρ is the set of desired POIs, and T_d_ is the real duration for day d. These two penalty functions are part of the objective function. The penalty function aims to tighten the calculation of MAUT, which represents user satisfaction. For example, if the user wants to visit 20 tourist attractions for 3 days, but the resulting itinerary only includes 15 tourist attractions, then the remaining five tours will be counted as a penalty $(pe{n_{POI}})$. In addition, the penalty related to the excess time of the travel duration per day has a function to tighten the calculation of the travel duration attribute. For instance, if there are two itineraries that both have a total travel duration of 26 h with a per day travel duration limit of 12 h, but the first itinerary is completed in 3 days (without any excess time per day) and the second itinerary is completed in 2 days with excess time on one of the days, then the better itinerary is the first one.

This research proposes an approach for optimal tour route recommendation in multi-day visits by considering the multi-criteria preferences of users. To accommodate the multi-criteria objective function, we use MAUT approach. MAUT is a method in decision theory that is used to evaluate and make decisions in situations involving multiple criteria or attributes. In MAUT, a utility function is used to measure how well a particular alternative meets the desired criteria. The value of this utility function is in the range $[0,1]$.

Suppose we define the set of attributes $A = \left\{ {{a_{1}},{a_{2}},{a_{3}}} \right\}$, with ${a_{1}} = \left( {{t_{{{\mathrm{tot}}}}}} \right),{a_{2}} = \bar r,\;{a_{3}} = f$. Since the values of each of these attributes have different scales, it is necessary to normalize them so that the evaluation of these attribute values is not biased. Suppose $v(a)$ is an attribute value for a POI, then we normalize it to get $v(a)^{\prime}$ (normalized result) as follows,

(22) $$v^{\prime} (a) = {{v(a) - v{{(a)}_{min}}} \over {v{{(a)}_{max}} - v{{(a)}_{min}}}}.$$

In this case, there are two types of attributes, *i.e*., lower better (*i.e*., $t$ dan $f$) and higher better (*i.e*., $\bar r$). In order for all attribute values to be compared, we need to transform an attribute value $a$ (denoted $s\left( {v(a)} \right)$) as follows,

(23) $$s(v^{\prime} (a)) = \left\{ {\matrix{ {{{v^{\prime} (a) - v^{\prime} ({a_{min}})} \over {v^{\prime} ({a_{max}}) - v^{\prime} ({a_{min}})}},} \hfill & {{\mathrm{higher\;better\;attribute}} } \hfill \cr {} \hfill & {} \hfill \cr {1 - {{v^{\prime} (a) - v^{\prime} ({a_{min}})} \over {v^{\prime} ({a_{max}}) - v^{\prime} ({a_{min}})}},} \hfill & {{\mathrm{lower\;better\;attribute}} } \hfill \cr } } \right..$$

In MAUT, each attribute has a weight value that represents its importance. In this study, we use DOI values to assign weights to each attribute of each POI, where ${{\mathrm{DO}}}{{{\mathrm{I}}}_{{a_{i}}}} \in [0,1]\;\forall {a_{i}} \in A$. For all attributes in *A*, we can formulate the utility function as follows,

(24) $$u\left( A \right) = {{\left( {\sum\nolimits_{{a_{j}} \in A} {s(v^{\prime} ({a_{j}})) \cdot DO{I_{{a_{j}}}}} } \right)} \over {\left( {\sum\nolimits_{{a_{j}} \in A} {DO{I_{{a_{j}}}}} } \right)}}\quad u(A) \in \left[ {0,1} \right]$$

where

$A = \{ {a_{1}},{a_{2}},{a_{3}}\}.$

This utility function is part of the objective function in this TOPTW optimization, combined with two penalty functions and a function to maximize the number of POIs in the itinerary. The resulting value of this objective function is the fitness value in KomoTrip. Based on Eq. (23), we transform the lower better attribute to support the “maximizing” objective function, so the objective function is as follows,

(25) $$ maximize \left( \frac{ \left( \sum_{a_j \in A} s(v'(a_j)) \cdot DOI_{a_j} \right) + s(v'(pen_{POI})) \, + s(v'(pen_{time}))  + s(v'(|P|)) }{ \left( \sum_{a_j \in A} DOI_{a_j} \right) + 3 } \right), \quad j \neq 0$$

**Figure 4:**
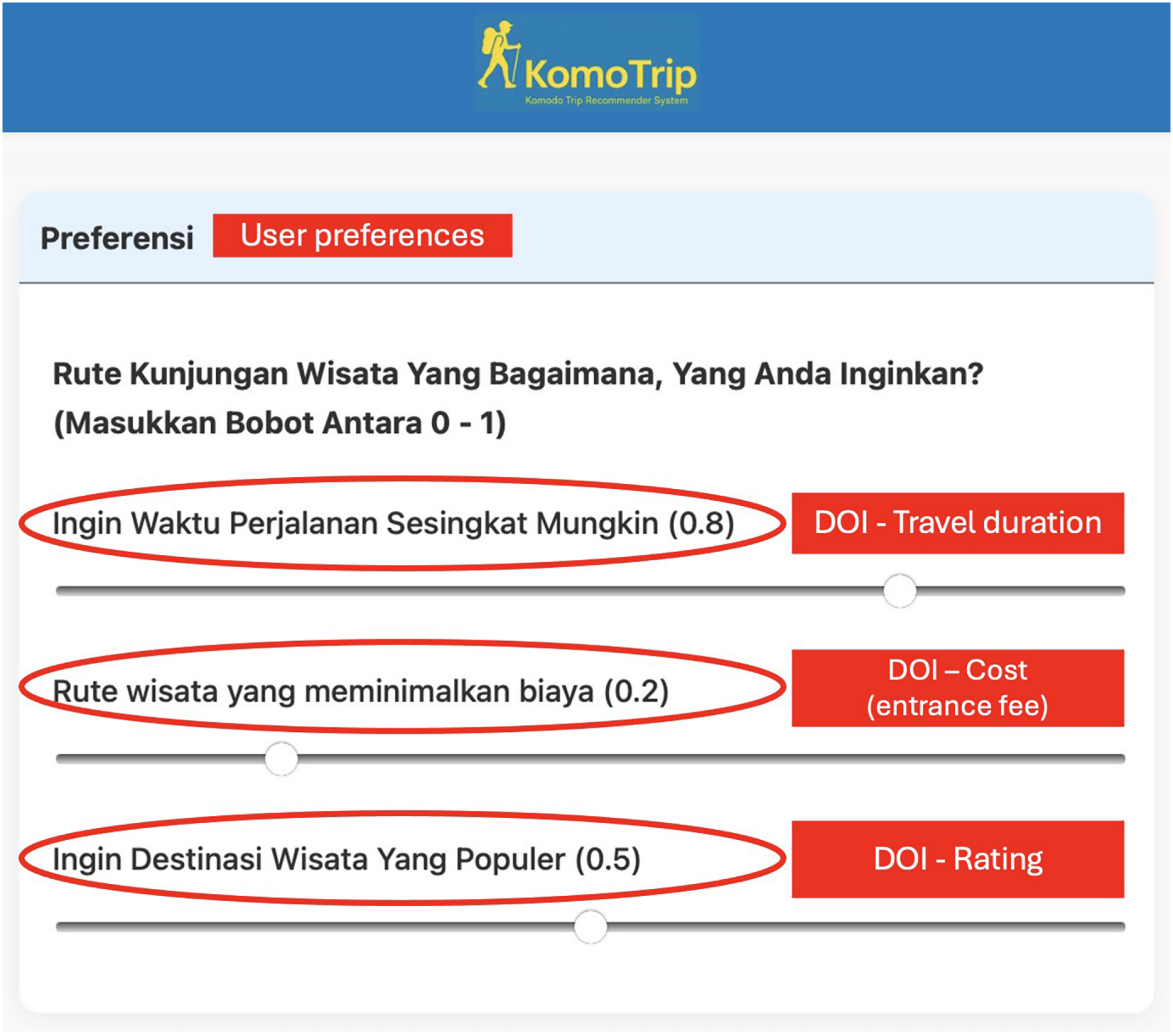
Screenshot of the website interface for users to input DOI values.

**Figure 5:**
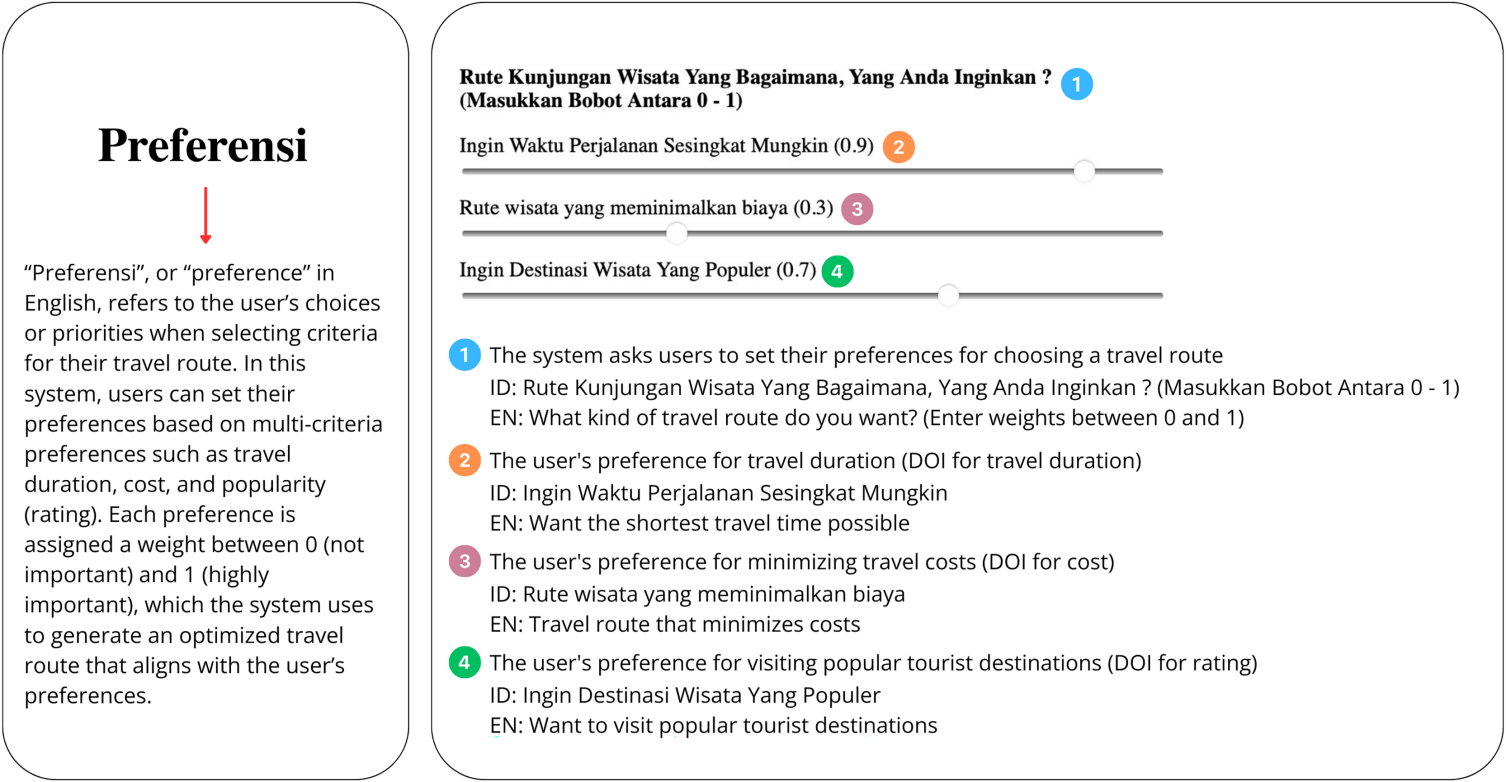
The English translation and explanation of DOI interface in Fig. 4.

The term $s\left( {v^{\prime} \left( {\left| P \right|} \right)} \right)$ in Eq. (25) shows that maximizing the number of itineraries is one part of the objective function. Figure 4 shows an example of user DOI entered into the system. The detailed explanation of each preference input is illustrated in Fig. 5.

### Algorithm for finding the optimal solution in KomoTrip

KomoTrip utilizes the Discrete Komodo Algorithm (DKA) (Jati et al., 2023) to recommend optimal multi-day routes, personalized based on user preferences. DKA is an extension of the Komodo Mlipir Algorithm (KMA), designed to solve combinatorial problems, particularly the Traveling Salesman Problem (TSP). Unlike the classic Komodo Mlipir Algorithm (KMA), the Discrete Komodo Algorithm (DKA) adopts a discrete solution representation and employs optimization operators inherently designed for discrete problems, thereby eliminating the need for the additional sorting step required in the original KMA. However, KomoTrip analogizes solving the optimal multi-day routes problem as a TOPTW solution. Some modifications are required, such as dividing the overall optimal route into daily segments and incorporating user-defined multi-attribute preferences into the optimization model. This section explains how KomoTrip operates, including the solution search algorithm based on the split_itinerary procedure (Algorithm 1) and the constraint modeling detailed in Section ‘Modeling for Constraint Function’.

The attraction movement refers to the process where Komodo A moves closer to Komodo B. In KomoTrip, the attraction movement is executed using an edge construction technique. Edge construction is a method for creating a new solution by making the solution of Komodo A resemble that of Komodo B. This technique forms edges from Komodo B onto Komodo A without disrupting the existing edges in either solution. The edge construction process begins by calculating the distance between Komodo A and Komodo B. Next, segmentation is carried out by dividing the travel itinerary into five segments based on the edge to be constructed. After segmentation, the final step is to create a new solution based on the distance and segmentation.

**Figure 6:**
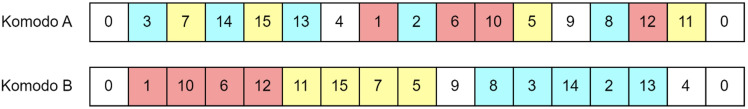
The distance between Komodo A and B is ten.

In this study, the distance between Komodo A and Komodo B is defined as the number of edges present in Komodo B but absent in Komodo A, as illustrated in Fig. 6. Figure 6 shows that there are ten edges present in Komodo B but not in Komodo A, *i.e*., edges $1{-}10,10{-}6,6{-}12,11{-}15,15{-}7,7{-}5,8{-}3,3{-}14,14{-}2,{{\mathrm{and}}}\;2{-}13$. The edges that start or end at node 0 (the hotel) are not considered in the distance calculation.

**Figure 7:**
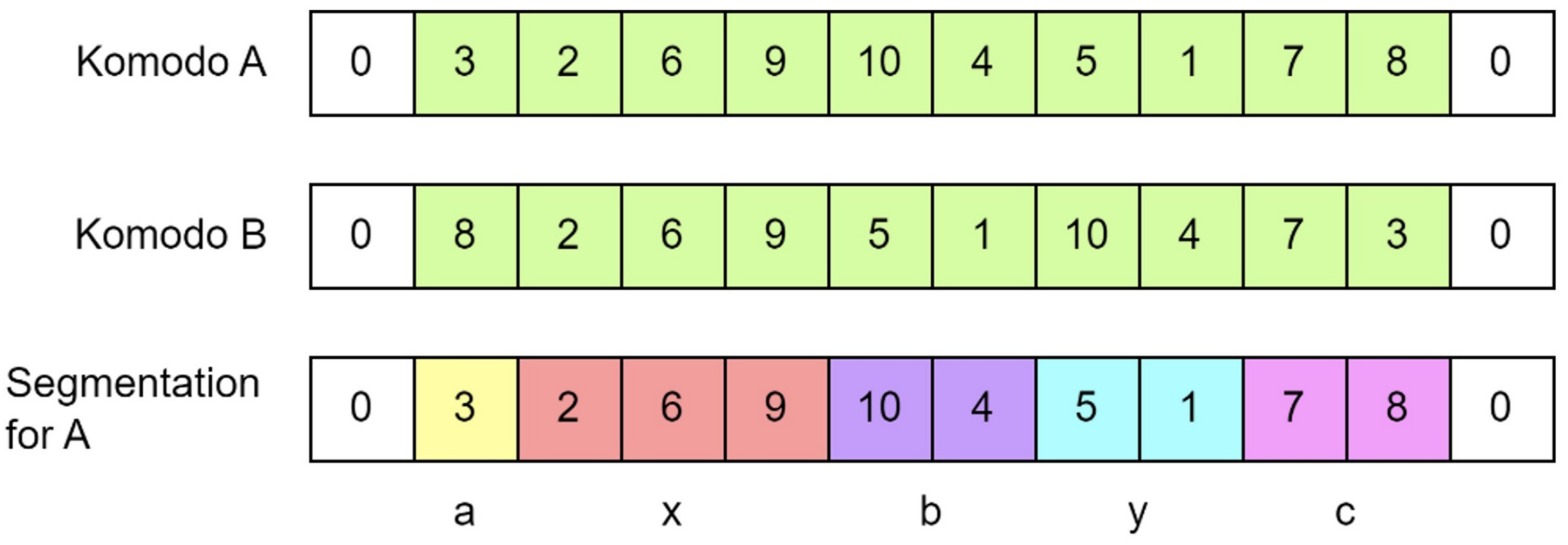
Segmentation for komodo A towards komodo B for edge 9–5 (attraction).

Figure 7 shows the segmentation process for edge $9{-}5$ between Komodo A and Komodo B (edge $9{-}5$ is present in Komodo B but absent in Komodo A). The segmentation process in edge construction divides the solution (Komodo) into a maximum of five segments. This process is carried out to make Komodo A resemble Komodo B (mimicking the movement of Komodo A approaching Komodo B). The process begins by randomly selecting an edge from Komodo B that does not exist in Komodo A; for example, in Fig. 7, the selected edge is $9{-}5$. Node 9 serves as point x, and node 5 as point y. Segment x is formed from point x and extends leftwards as long as the edges remain in Komodo B. Segment y is formed from point y and extends rightwards as long as the edges remain in Komodo B. Subsequently, segments a, b, and c are created in the remaining empty spaces (those not yet segmented).

**Figure 8:**
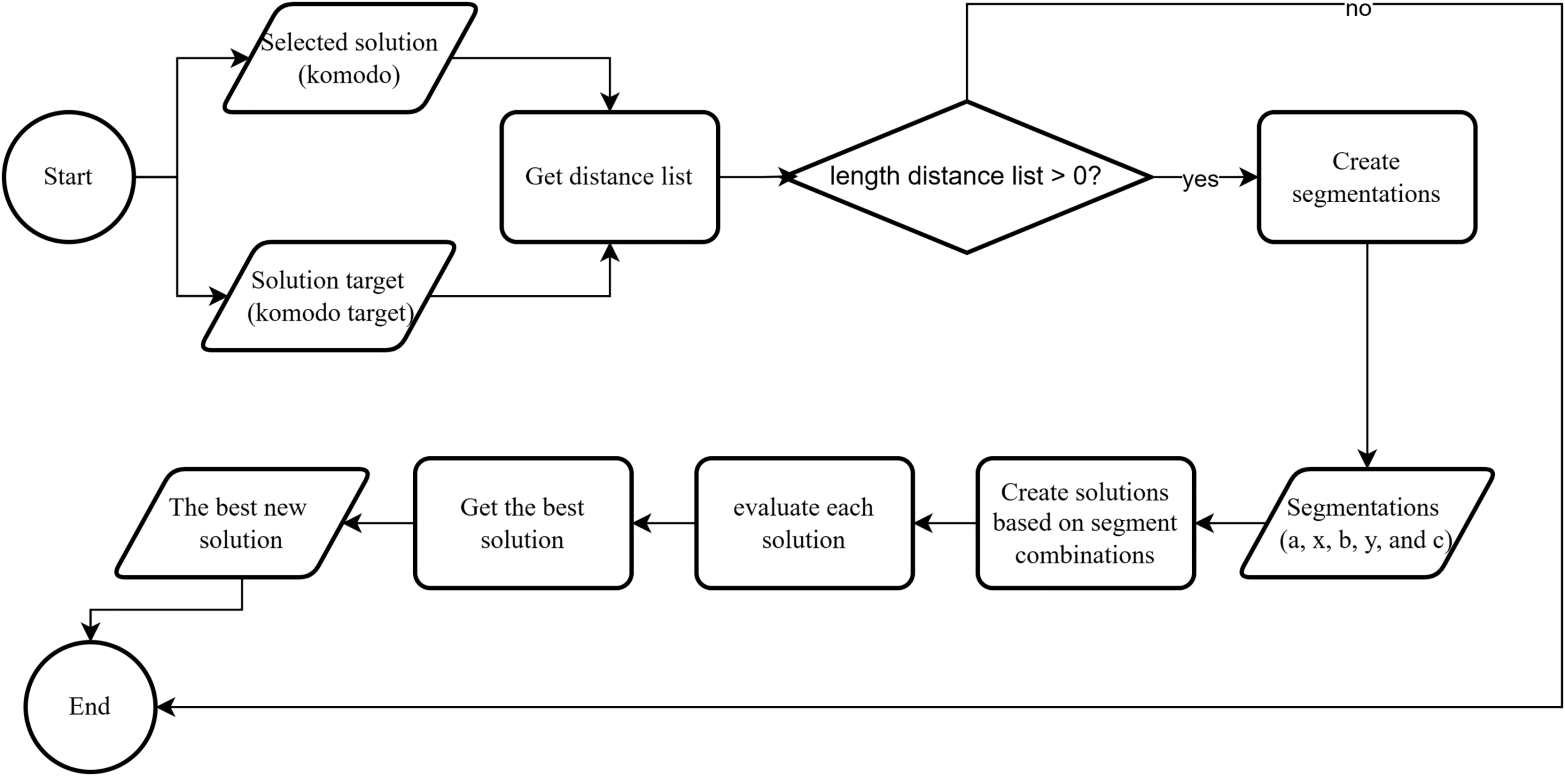
The edge construction flowchart.

Figure 8 shows an overview of the edge construction mechanism in our KomoTrip. Algorithm 2 then details the procedure, which takes input in the form of a Komodo and a target Komodo. The Komodo refers to the one that aims to approach the target Komodo. If Komodo A seeks to approach Komodo B, the process begins by creating a distance list, which is a list of edges present in Komodo B but not in Komodo A (line 2). Next, if Komodo A and B have a distance, one edge is selected from the distance list, and segmentation is performed based on Fig. 7 (lines 3 to 5). Subsequently, several new Komodos or possible solutions are generated based on the rules outlined in lines 6 to 9 of Algorithm 2. Itinerary separation is conducted in lines 10–12. Each new solution is then evaluated by calculating the objective function to accommodate the multi-criteria nature of user preferences (line 13). Finally, the best result is selected as the output of Algorithm 2.

**Algorithm 2:** Edge construction in KomoTrip.

1: **Function** edge_construction(komodo, komodo_target)
2: distance_list ← edge distance between komodo and komodo_target
3: **if** length(distance_list) *>* 0 **then**
4: choose a random edge from distance_list
5: create segment a, segment x, segment b, segment y, and segment c
6: result[1] ← segment a + segment b + segment x + segment y + segment c
7: result[2] ← segment a + segment b + reverse(segment y) + reverse(segment x) + segment c
8: result[3] ← segment a + reverse(segment y) + reverse(segment x) + segment b + segment c
9: result[4] ← segment a + segment x + segment y + segment b + segment c
10: **for** i in results **do**
11: result[i] ← split_itinerary(result[i])
12: **end for**
13: evaluate each result using objective function, Eq. (24)
14: best_result ← the best result based on objective function using Eq. (24)
15: best_operator ← best_result
16: **else**
17: best_operator ← Komodo {komodo and komodo_target have no differences}
18: best_operator ← split_itinerary(best_operator)
19: **end if**
20: **return** best_operator

The distraction movement is a process where Komodo A moves away from Komodo B. In DKA, the distraction movement is performed using the edge destruction technique. Edge destruction works by removing an edge in Komodo A that is also present in Komodo B, thereby increasing the distance between Komodo A and Komodo B. The edge destruction process begins by calculating the similarity between Komodo A and Komodo B, performing segmentation on Komodo A, and generating new solutions based on their similarity and segmentation.

**Figure 9:**
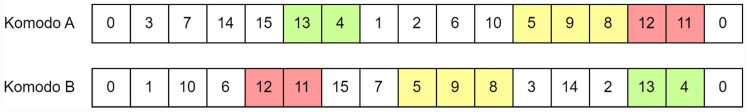
The similarity between komodo A and komodo B is four.

In this study, the similarity between two solutions (Komodos) is defined as the number of edges present in both solutions. Figure 9 illustrates the calculation of similarity between Komodo A and Komodo B. The similarity between Komodo A and Komodo B is four, as indicated by the edges $5{-}9,9{-}8,12{-}11,{{\mathrm{and}}}\;13{-}4$.

**Figure 10:**
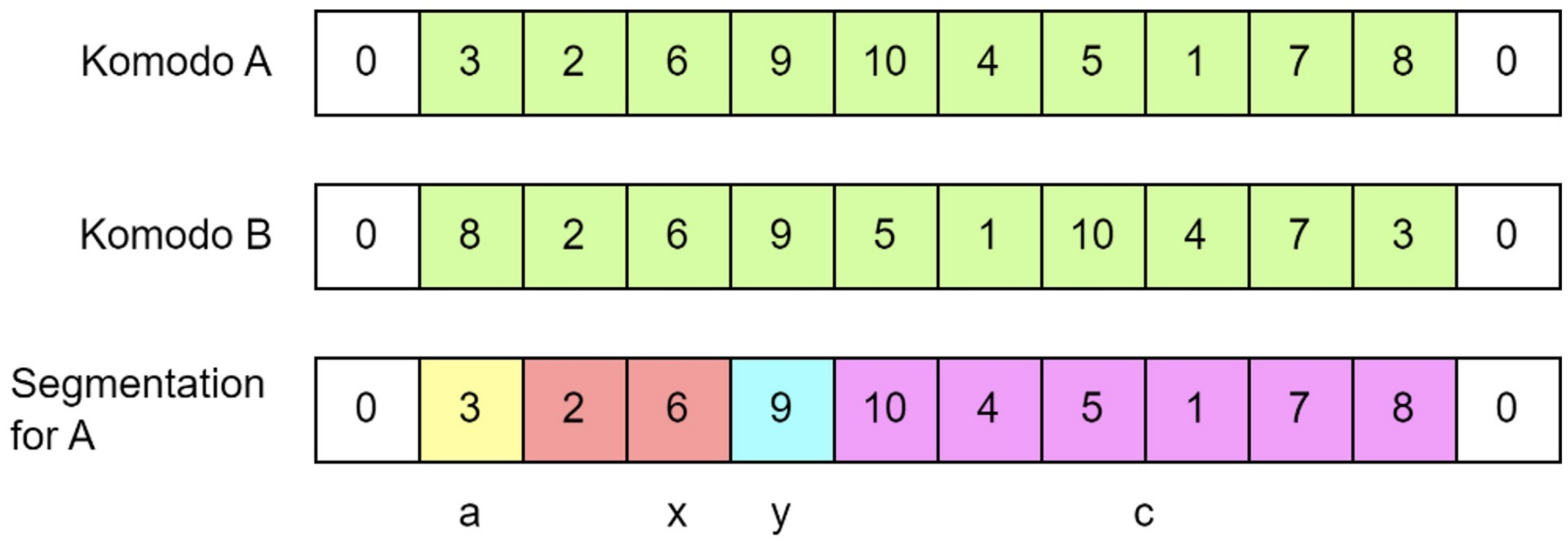
Segmentation for komodo A towards komodo B for edge 6–9 (distraction).

The segmentation process in the distraction movement, as shown in Fig. 10, is similar to the segmentation process in the attraction movement. The key difference is that in the segmentation for distraction movement, the selected edge is one that exists in both Komodo A and Komodo B. This results in the segmentation for distraction movement having a maximum of four segments, without segment b (*i.e*., there is no segment between segment x and segment y).

**Figure 11:**
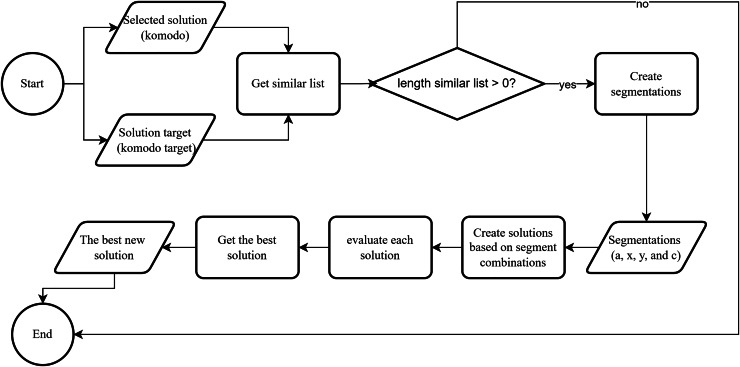
The edge destruction flowchart.

Edge destruction is outlined in Algorithm 3 in our KomoTrip, with an overview of the overall process illustrated in Fig. 11. Similar to the edge construction process in Algorithm 2, edge destruction in Algorithm 3 takes two inputs: the Komodo and the target Komodo. Edge destruction represents the movement of Komodo A away from Komodo B. The process begins by identifying the edges present in both Komodo A and Komodo B (line 2). Next, one similar edge is randomly selected, and segmentation is performed for the distraction process (lines 3 to 5). Subsequently, new Komodos or potential solutions are generated based on the rules defined in lines 6 to 11. Itinerary separation is conducted in lines 11–14. Each new solution is then evaluated using the objective function (line 15). Finally, the best solution is selected as the final output (lines 16 to 22).

**Algorithm 3:** Edge destruction in KomoTrip.

1: **Function** edge_destruction (komodo, komodo_target)
2: similar_list ← list of similar edges between komodo and komodo_target
3: **if** length (similar_list) *>* 0 **then**
4: choose a random edge from similar_list
5: create segment a, segment x, segment y, and segment c
6: result[1] ← segment x + segment a + segment y + segment c
7: result[2] ← segment a + segment y + segment x + segment c
8: result[3] ← segment a + reverse (segment x) + reverse (segment y) + segment c
9: result[4] ← segment a + reverse (segment x) + segment y + segment c
10: result[5] ← segment a + segment x + reverse(segment y) + segment c
11: result[6] ← segment a + segment x + segment y + segment c
12: **for** i in results **do**
13: result[i] ← split_itinerary(result[i])
14: **end for**
15: evaluate each result using objective function, Eq. (24)
16: best_result ← the best result based on objective function using Eq. (24)
17: best_operator ← best_result
18: **else**
19: best_operator ← Komodo {komodo and komodo_target have no similarity}
20: best_operator ← split_itinerary(best_operator)
21: **end if**
22: **return** best_operator

**Figure 12:**
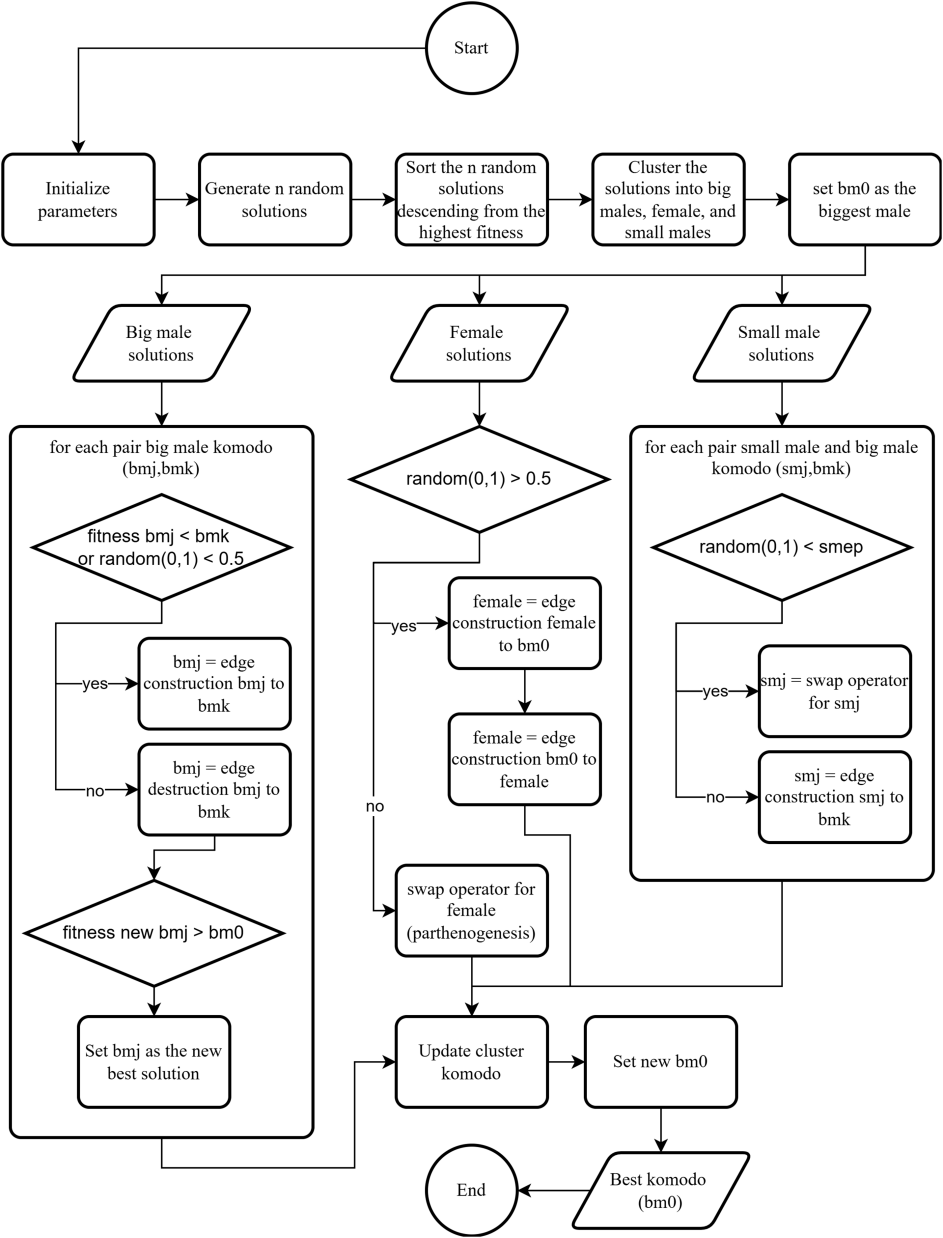
The optimal search flowchart.

In addition to attraction and distraction movements, there is the exploration movement in DKA. The exploration movement replaces the mlipir movement of small male Komodos and the parthenogenesis of female Komodo. In this study, the exploration movement is performed using a swap operator. The swap operator is a process that swaps the positions of two randomly selected nodes. Attraction movement is used by big males, females, and small males. Distraction movement is used by big males, while exploration movement is used by females and small males. These three movements are utilized in KomoTrip, as shown in Algorithm 4 and further depicted in Fig. 12.

**Algorithm 4:** Search optimal solution in KomoTrip.

1: **Function** optimal_search(*n, p, smep, N*)
2: { $n,p,smep,{\mathrm{and}} \;N$ are the DKA parameters that need to be initialized first}
3: generate *n* random solutions
4: sort the *n* random solutions descending from the biggest fitness value
5: cluster the solutions into big males (*bm*), female (*f*), and small males (*sm*) { $b{m_{0}}$ is the biggest male}
6: best_solution $\leftarrow b{m_{0}}$
7: **for** $i \leftarrow 1$ to *N* **do**
8: {big males movement}
9: **for all** *j* in *bm* **do**
10: new_bm $\leftarrow \left[ \, \right]$
11: **for all** *k* in *bm* **do**
12: **if** fitness $b{m_{j}} \lt$ fitness $b{m_{k}}$ or random(0,1) < 0.5 **then**
13: new_solution ← edge_construction( $b{m_{j}},b{m_{k}}$) {attraction}
14: **else**
15: new_solution ← edge_destruction( $b{m_{j}},b{m_{k}}$) {distraction}
16: **end if**
17: append new_solution to new_bm
18: **if** $b{m_{j}} = b{m_{0}}$ **then**
19: **if** fitness $b{m_{j}} \gt$ fitness the best solution in new_bm **then**
20: $b{m_{j}} \leftarrow$ the best solution in new_bm
21: **end if**
22: **else**
23: $b{m_{j}} \leftarrow$ the best solution in new_bm
24: **end if**
25: **end for**
26: **end for**
27: {female movement}
28: **if** random(0,1) < 0.5 **then**
29: new_solution1 ← edge_construction( $f,b{m_{0}}$)
30: new_solution2 ← edge_construction( $b{m_{0}},f$)
31: $fm \leftarrow$ the best solution between new_solution1 and new_solution2
32: **else**
33: $fm \leftarrow$ swap_operator(*fm*) {exploration (parthenogenesis)}
34: **end if**
35: {small males movement}
36: **for all** *j* in *sm* **do**
37: new_sm $\leftarrow \left[ \, \right]$
38: **for all** *k* in *bm* **do**
39: **if** random(0,10) $< smep$ **then**
40: new_solution ← swap_operator( $s{m_{j}}$) {exploration (mlipir)}
41: **else**
42: new_solution ← edge_construction( $s{m_{j}},b{m_{k}}$) {attraction}
43: **end if**
44: append new_solution to new_sm
45: $s{m_{j}} \leftarrow$ the best solution in new_sm
46: **end for**
47: **end for**
48: update cluster komodo based on fitness {get new big males, female, and small males members}
49: **if** $b{m_{0}}$ outperforms best_solution **then**
50: best_solution $\leftarrow b{m_{0}}$
51: **end if**
52: **end for**
53: **return** best_solution

DKA has the parameters $n$, $p$, $smep$, and *N*. The parameter $n$ represents the number of Komodos generated for the optimization process, $p$ represents the percentage of big males, $smep$ represents the probability of small male exploration (ranging from 0 to 10), and *N* denotes the maximum number of iterations. The initial step is to generate $n$ random solutions (Komodos), as shown in line 3 of Algorithm 4. Next, the generated Komodos are grouped into big males, females, and small males (lines 4 to 5). Lines 7 to 26 describe the movement of big males, utilizing edge construction and edge destruction to find new solutions. New solutions are obtained by comparing the fitness values (MAUT) between the new and existing solutions. Lines 27 to 33 detail the female movement, which uses edge construction and swap operators to discover new solutions. Lines 33 to 47 cover the movement of small males. Subsequently, the Komodo cluster is updated to identify new members of big males, females, and small males (line 48). Finally, the best solution from all iterations is selected as the output of Algorithm 4 (lines 49 to 53).

**Figure 13:**
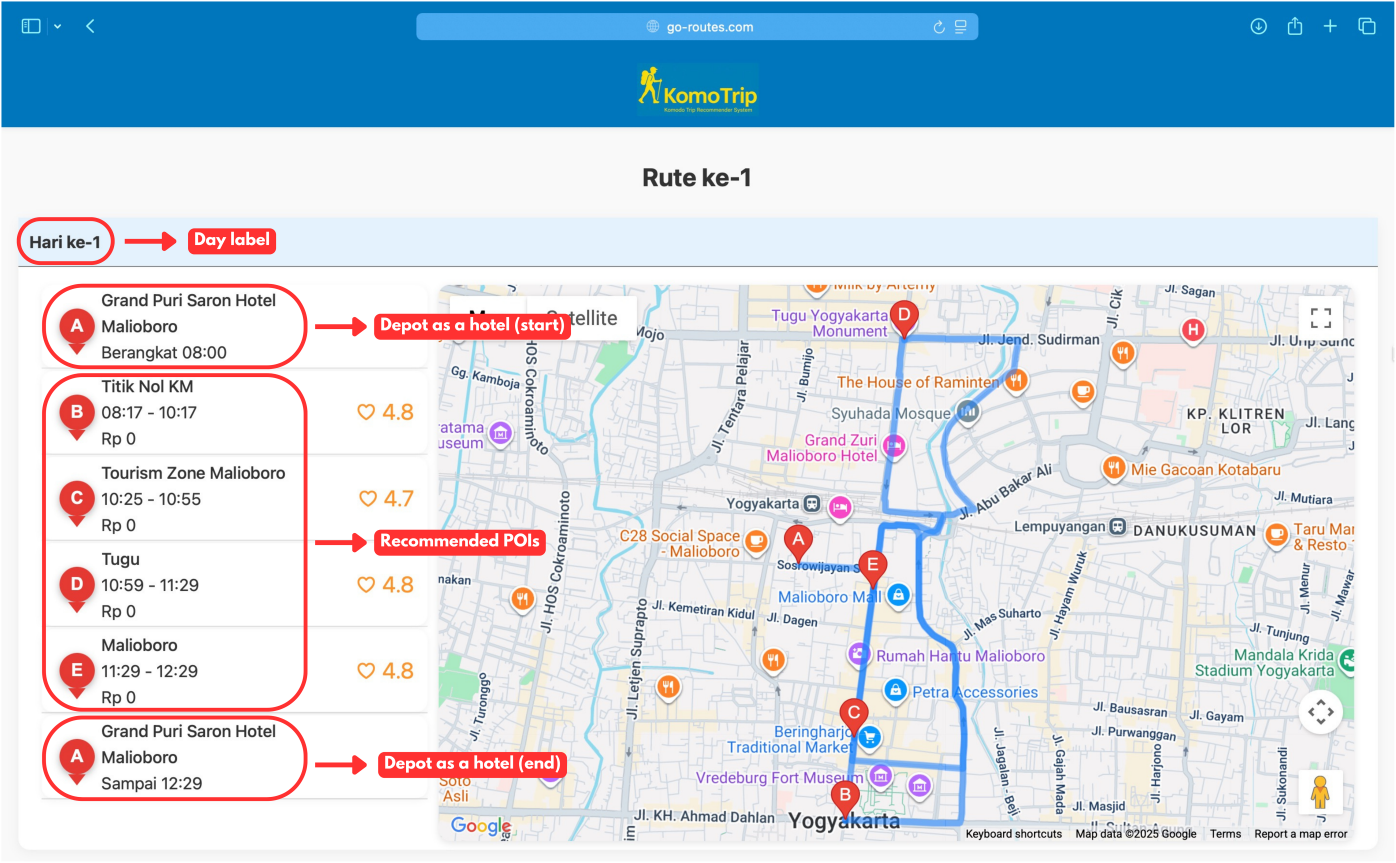
The example of route recommendation generated by KomoTrip for day-1. Departing from Grand Putri Saron Hotel, the recommended itinerary: Hotel $\to$ Titik Nol KM $\to$ Tourism Zone Malioboro $\to$ Tugu $\to$ Malioboro $\to$ Hotel. Map data (©) 2025 Google.

**Figure 14:**
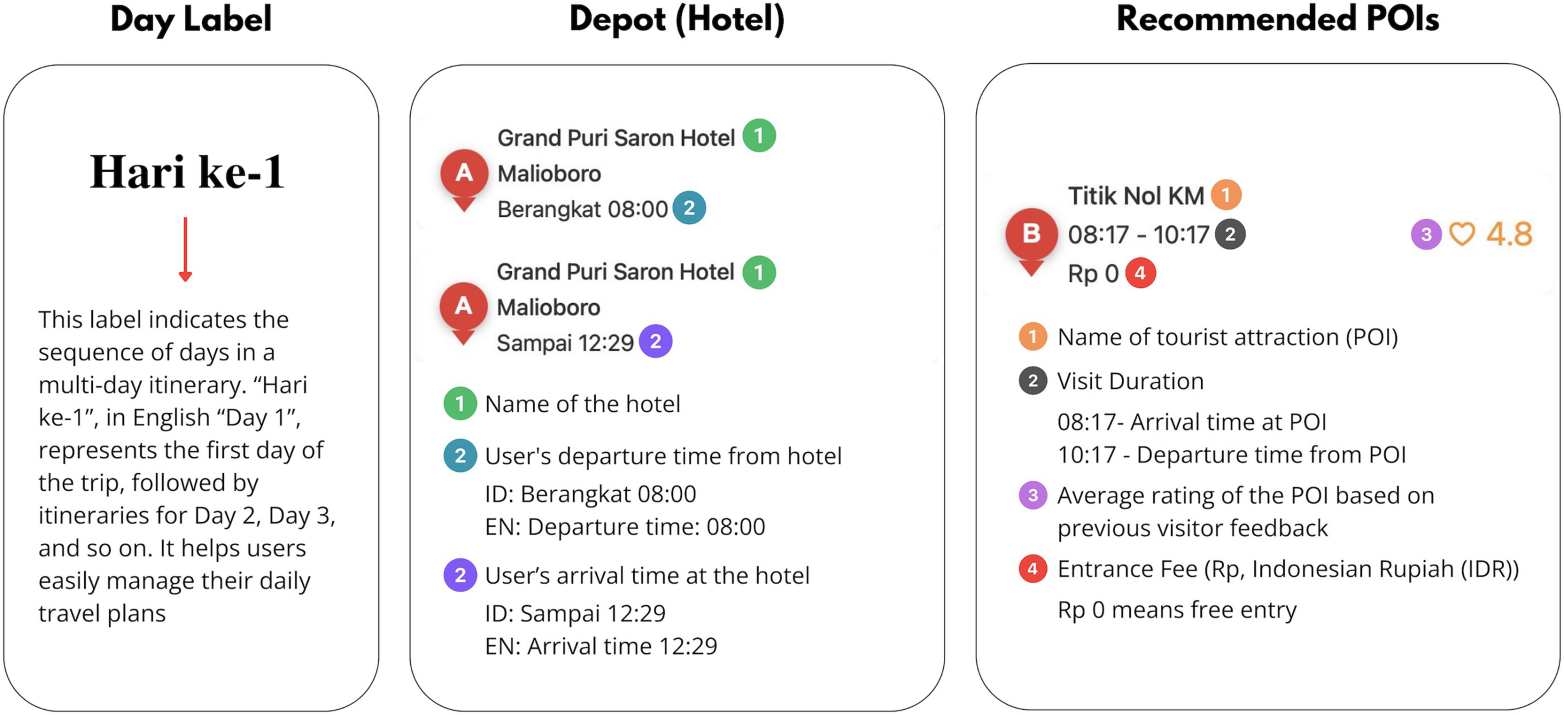
The English translation and explanation of the itinerary interface in Fig. 13.

Figure 13 depicts an example of the optimal route recommendation for day 1, generated by KomoTrip in our developed system (https://go-routes.com). The route starts and ends at the depot (hotel), marked as A, and includes several tourist attractions (POIs) along the route. The detailed breakdown of these system elements, specifically the Day Label, Depot (Hotel), and Recommended POIs highlighted in red, is presented in Fig. 14. This figure explains the structure and information shown in each section of the itinerary interface.

## Experimental results

For evaluation, we conduct a series of experiments to analyze the performance of the proposed method, KomoTrip. Prior to the experiments, we perform hyperparameter tuning for KomoTrip. We conduct initial experiments to evaluate the performance of KomoTrip’s basic algorithm (we call as original KMA), before a series of tests that focus on the performance of KomoTrip. These experiments aim to understand how original KMA works in solving discrete problems by comparing it with KomoTrip (proposed model) and state-of-the-art models/algorithms. The comparison algorithms for evaluating the performance of original KMA and KomoTrip are SA (Tlili & Krichen, 2021), ACS and ACO (Verbeeck, Vansteenwegen & Aghezzaf, 2017), TS (Sylejmani, Dorn & Musliu, 2012), MOGA (Moosavi Heris et al., 2022), DPSO (Yu et al., 2019), BSO (Hendrawan, Baizal & Wulandari, 2024), WOA-VNS (Ananda, Baizal & Wulandari, 2024), ILS and its hybridizations (ILS-SA, ILS-TS, and ILS-SA-TS) (Sylejmani et al., 2024), and MGA (Pérez-Cañedo et al., 2024).

The original KMA evaluation serve as the basis for assessing whether the KomoTrip development can provide better performance compared to the original KMA and other algorithms. The evaluations are conducted in three scenarios, as follows:

(1) Scenario 1: General performance testing to analyze the overall performance of the original KMA and KomoTrip compared to other algorithms, especially for handling multi-attribute preference cases.
(2) Scenario 2: Testing on various Degree of Interest (DOI) combinations to assess the performance of the original KMA and KomoTrip algorithms in different user preference scenarios.
(3) Scenario 3: Testing robustness to changes in problem dimension and size, by looking at performance trends through changes in the number of Points of Interest (POIs).

The two main metrics used in this experiment are fitness value and running time. To test whether each result is significantly different, we use a two-tailed Wilcoxon rank-sum test with a significance level of $(\sigma ) = 0.05$. We present the results of the Wilcoxon rank-sum test in a table consisting of the *p*-value accompanied by the codes 1, 0, −1. The codes 1, 0, −1 represent that KMA or KomoTrip performs significantly better, not significantly different, or significantly worse, respectively, than the comparison algorithm. All experiments are implemented in Python on a 12th Gen Intel Core i7-12700H 2.3 GHz processor with 16 GB RAM.

In addition to the three evaluation scenarios mentioned above, we also conduct sensitivity analysis and convergence evaluation in the Hyperparameter Tuning section. To strengthen the validity of KomoTrip’s performance, we also perform benchmarking against other algorithms through a public dataset (Solomon dataset (https://www.mech.kuleuven.be/en/cib/op)), in a separate section.

### Hyperparameter tuning

**Figure 15:**
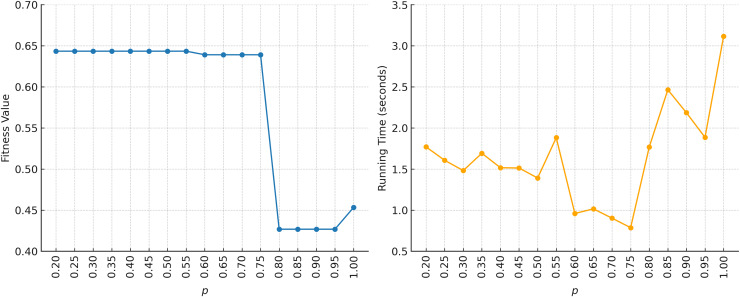
Sensitivity analysis of KomoTrip on parameter *p* with *smep = 5*.

**Figure 16:**
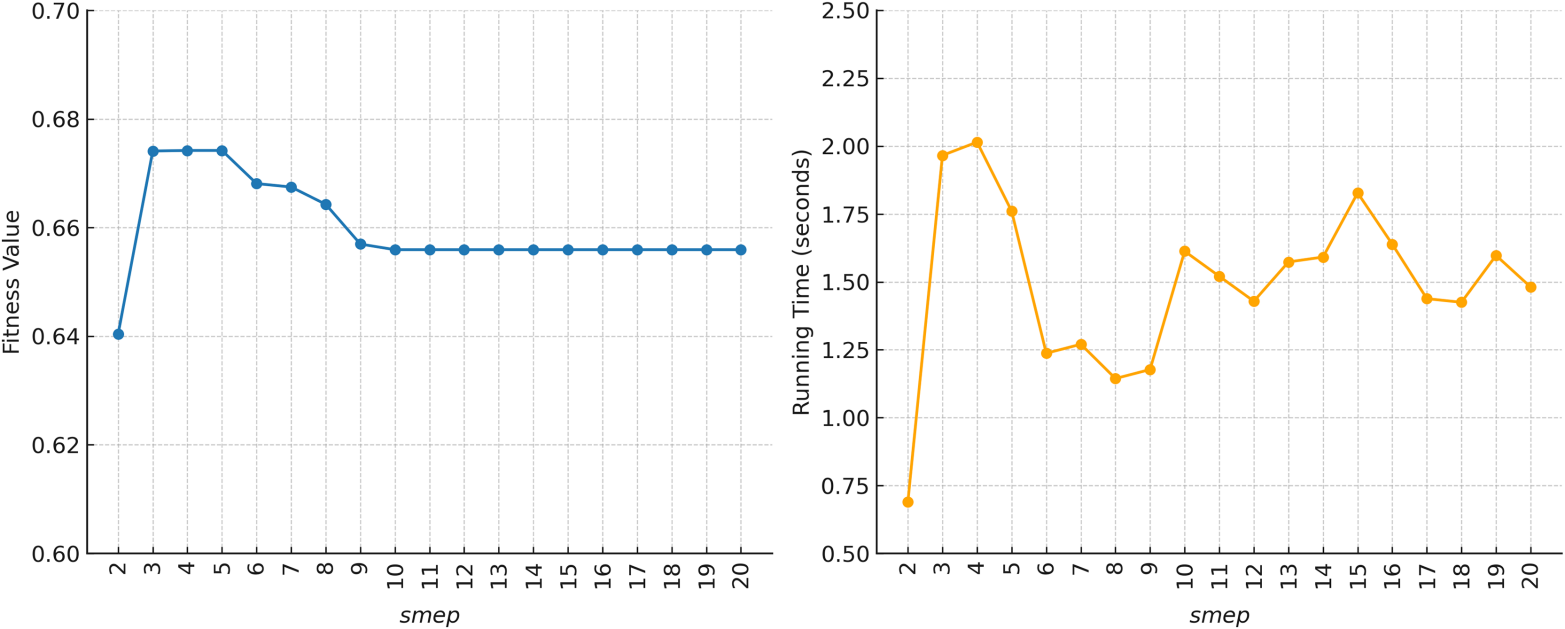
Sensitivity analysis of KomoTrip on parameter *smep* with *p* = 0.5.

KomoTrip has two main hyperparameters to tune, *i.e*., the number of iterations $(N)$ and the number of komodos $(n)$. Meanwhile, we use parameters $p = 0.5$ and $smep = 5$, referring to the original KMA setting (Suyanto, Ariyanto & Ariyanto, 2022). The results of the sensitivity test for these parameter settings are shown in Figs. 15 and 16. Based on Fig. 15, for $smep = 5$, the fitness value tends to remain stable at $p = 0.5$– $0.75$, then decreases within the range of $p = 0.8$– $1.0$. Meanwhile, the running time tends to remain stable in the range of $p = 0.2$– $0.55$. Outside this range, the running time becomes more fluctuating. The parameter settings of $p = 0.5$ and $smep = 5$ appear consistent, as we prioritize fitness achievement over running time. For example, the highest fitness is observed in the range of $p = 0.2$– $0.55$, while the fastest running time occurs at $p = 0.5$.

In Fig. 16, for $p = 0.5$, fitness values tend to be stable within the ranges of $smep = 9$– $20$ and $smep = 3$– $5$. Meanwhile, for $p = 0.5$, running time also tends to remain stable within $smep = 10$– $20$. The choice of $p = 0.5$ and $smep = 5$ again appears consistent, as we prioritize fitness gains over running time in the parameter selection. The highest fitness is found in the range of $smep = 3$– $5$, with the fastest running time occurring at $smep = 5$. However, the selection of optimal parameters is not solely based on this sensitivity test, but rather obtained through hyperparameter tuning, as previously conducted in Suyanto, Ariyanto & Ariyanto (2022). Overall, the sensitivity test indicates that changes in the $smep$ value do not significantly affect the fitness value, which stays around 0.6.

The number of iterations is determined based on convergence analysis, which is performed by running the process until it reaches a convergent state. In this study, the solution is considered to have converged if the best fitness value does not change for 20 consecutive iterations.

**Figure 17:**
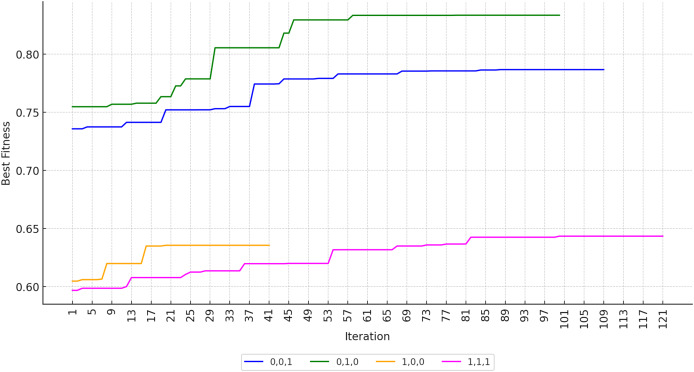
Best fitness convergence of KomoTrip over iterations.

Figure 17 shows that KomoTrip reaches convergence in less than 100 iterations on average. In the scenario that only considers the travel duration attribute, KomoTrip requires fewer iterations to reach convergence, although the resulting fitness value is relatively low. This shows that KomoTrip is not optimal in maximizing travel duration, but is able to optimize other attributes better.

The number of komodos is determined through experiments with a configuration of 30 POIs, a tour duration of 3 days, and equal DOI weights for all attributes (travel duration, fare, and rating) set to (1, 1, 1). In this research, we target to develop an algorithm that is fast while still producing an acceptable solution. Based on the test results summarized in Table 1, the number of komodos that gives the best result is 5 komodos.

**Table 1:** Configuration of the number of komodos for hyperparameter tuning. The best values are highlighted in bold.

*n*	Fitness value	Running time
**5**	**0.6434**	**2.2668**
10	0.6667	6.9904
15	0.6647	17.5756
20	0.6739	60.8990

### Scenario 1: general performance

The purpose of this experiment is to evaluate the performance of each preference criterion, as well as the aggregation of multi-criteria user preferences. We repeat the experiment for each method 50 times. Each experiment consists of 30 POIs for a three-day trip. There are four cases related to the combination of DOI values in this experiment, *i.e*.,: (1) all DOIs are set to 1 (aggregation of all preference criteria), (2) only travel duration DOI is 1, (3) only cost DOI is 1, and (4) only rating DOI is 1, while other DOI values are set to 0.

#### Original KMA

In case 1, where the DOI value is set to 1, the fitness value and running time generated by the original KMA and other comparison methods are presented in Table 2. To evaluate the significance of the performance difference in these values, the Wilcoxon rank-sum test results are shown in Table 3.

**Table 2:** Fitness values and running times of the original KMA *vs*. other methods in case 1.

Method	Fitness value	Running time
**KomoTrip (Proposed method)**	0.6408	0.4629
Original KMA	0.6563	5.0643
ACS	0.6593	3.7462
ACO	0.6399	2.6912
SA	0.6582	0.9717
DPSO	0.6474	0.6048
TS	0.6402	0.6650
WOA-VNS	0.6405	0.4147
MOGA	0.6309	0.7422
BSO	0.5529	0.3784
ILS	0.5912	2.5352
ILS-SA	0.6151	8.5597
ILS-TS	0.5906	11.1283
ILS-SA-TS	0.6153	23.2250
MGA	0.6557	0.5403

**Table 3:** Statistical test results for the original KMA *vs*. other methods in case 1.

Comparative method	*p*-value	
	Fitness value	Running time
**KomoTrip (Proposed method)**	0.0011 (1)	6.9E−18 (−1)
ACS	0.4863 (0)	0.0050 (−1)
ACO	0.0016 (1)	1.1E−08 (−1)
SA	0.6148 (0)	6.9E−18 (−1)
DPSO	0.0441 (1)	6.9E−18 (−1)
TS	0.0012 (1)	6.9E−18 (−1)
WOA-VNS	0.0009 (1)	6.9E−18 (−1)
MOGA	2.3E−06 (1)	6.9E−18 (−1)
BSO	9.3E−18 (1)	6.9E−18 (−1)
ILS	1.5E−12 (1)	1.6E−09 (−1)
ILS−SA	4.8E−08 (1)	2.3E−06 (1)
ILS-TS	9.2E−14 (1)	3.8E−11 (1)
ILS-SA-TS	1.9E−08 (1)	2.9E−17 (1)
MGA	0.8795 (0)	6.9E−18 (−1)
Wilcoxon (0)	3	0
Wilcoxon (1)	11	3
Wilcoxon (−1)	0	11

Based on 3, the fitness value generated by the original KMA does not show a significant difference when compared to ACS, SA, and MGA. On the other hand, the original KMA shows an advantage in terms of fitness value compared to the majority of comparison algorithms, such as KomoTrip, ACO, DPSO, TS, WOA-VNS, MOGA, BSO, ILS, ILS-SA, ILS-TS, and ILS-SA-TS. However, in terms of running time, the original KMA has a longer execution time than most comparison algorithms, except ILS-SA, ILS-TS, and ILS-SA-TS. The relatively high computational cost of ILS arises from its sequential nature and the extensive evaluation of candidate solution neighborhoods. When hybridized with TS, the complexity further increases since the algorithm must examine a larger set of neighbors to identify the best admissible (non-tabu) move. In contrast, the ILS–SA hybrid introduces additional overhead through the acceptance criterion of SA and allows a broader exploration by evaluating a greater number of inferior solutions.

The analysis continues for case 2, with travel duration set to be the top priority, without considering cost and rating. The results of this second scenario are shown in Table 4. In Table 5, we show the results of the Wilcoxon rank-sum test to see if the values in Table 4 are significantly different. From Table 5, we can see that, when travel duration is the top priority, the fitness values of the original KMA are not significantly different from ACS, ACO, SA, and MGA. Meanwhile, for the majority, the original KMA is significantly superior to the comparison algorithms (KomoTrip, DPSO, TS, WOA-VNS, MOGA, BSO, ILS, ILS-SA, ILS-TS, and ILS-SA-TS). On the other hand, in terms of running time, the original KMA again shows less efficient performance than most of the comparison algorithms, as in the previous case (with ILS-TS and ILS-SA-TS remaining the exceptions, while the difference with ILS-SA is no longer statistically significant).

**Table 4:** Fitness values and running times of the original KMA *vs*. other methods in case 2.

Method	Fitness value	Running time
**KomoTrip (Proposed method)**	0.6379	0.4369
Original KMA	0.6530	6.3568
ACS	0.6536	3.8648
ACO	0.6524	2.6745
SA	0.6519	0.9470
DPSO	0.6436	0.6203
TS	0.6413	0.6482
WOA-VNS	0.6348	0.4307
MOGA	0.6299	0.7139
BSO	0.5860	0.3421
ILS	0.6129	2.5811
ILS-SA	0.6242	7.1629
ILS-TS	0.6127	11.2673
ILS-SA-TS	0.6278	20.7614
MGA	0.6460	0.5135

**Table 5:** Statistical test results for the original KMA *vs*. other methods in case 2.

Comparative method	*p*-value	
	Fitness value	Running time
**KomoTrip (Proposed method)**	0.0013 (1)	6.9E−18 (−1)
ACS	0.7907 (0)	7.9E−08 (−1)
ACO	0.9862 (0)	1.2E−14 (−1)
SA	0.9067 (0)	6.9E−18 (−1)
DPSO	0.0296 (1)	6.9E−18 (−1)
TS	0.0161 (1)	6.9E−18 (−1)
WOA-VNS	0.0001 (1)	6.9E−18 (−1)
MOGA	1.3E−06 (1)	6.9E−18 (−1)
BSO	7.9E−15 (1)	6.9E−18 (−1)
ILS	3.5E−09 (1)	7.8E−14 (−1)
ILS-SA	5.7E−07 (1)	0.0637 (0)
ILS-TS	3.5E−10 (1)	9.3E−09 (1)
ILS-SA-TS	1.4E−06 (1)	4.1E−16 (1)
MGA	0.1496 (0)	6.9E−18 (−1)
Wilcoxon (0)	4	1
Wilcoxon (1)	10	2
Wilcoxon (−1)	0	11

After analyzing the previous two cases, case 3 turns to the priority of the DOI cost value to measure the algorithm’s ability to minimize the travel cost. In this case, travel duration and rating are not taken into account. The fitness value and running time results for each algorithm in this scenario are listed in Table 6, while Table 7 contains the Wilcoxon rank-sum test results to evaluate the significance of performance differences between algorithms based on these two metrics.

**Table 6:** Fitness values and running times of the original KMA *vs*. other methods in case 3.

Method	Fitness value	Running time
**KomoTrip (Proposed method)**	0.8000	0.4069
Original KMA	0.8262	4.6247
SA	0.8257	0.9577
ACS	0.8251	3.6435
DPSO	0.8149	0.5153
TS	0.8071	0.6635
WOA-VNS	0.8039	0.3659
MOGA	0.7946	0.7674
ACO	0.7869	2.8034
BSO	0.6614	0.1938
ILS	0.7292	2.1644
ILS-SA	0.7554	6.8591
ILS-TS	0.7232	7.5429
ILS-SA-TS	0.7665	18.0942
MGA	0.8090	0.3534

**Table 7:** Statistical test results for the original KMA *vs*. other methods in case 3.

Comparative method	*p*-value	
	Fitness value	Running time
**KomoTrip (Proposed method)**	4.7E−06 (1)	6.9E−18 (−1)
SA	0.8958 (0)	6.9E−18 (−1)
ACS	0.7277 (0)	0.0264 (−1)
DPSO	0.0045 (1)	6.9E−18 (−1)
TS	5.7E−05 (1)	6.9E−18 (−1)
WOA-VNS	1.3E−05 (1)	6.9E−18 (−1)
MOGA	3.9E−08 (1)	1.3E−17 (−1)
ACO	2.8E−10 (1)	3.1E−07 (−1)
BSO	6.9E−18 (1)	6.9E−18 (−1)
ILS	2.0E−16 (1)	3.8E−11 (−1)
ILS-SA	4.1E−12 (1)	2.9E−05 (1)
ILS-TS	6.5E−17 (1)	5.2E−06 (1)
ILS-SA-TS	1.2E−10 (1)	1.0E−16 (1)
MGA	0.0004 (1)	6.9E−18 (−1)
Wilcoxon (0)	2	0
Wilcoxon (1)	12	3
Wilcoxon (−1)	0	11

The results in Table 7 show that the original KMA produces higher fitness values compared to the majority of the comparison algorithms (KomoTrip, DPSO, TS, WOA-VNS, MOGA, ACO, BSO, ILS, ILS-SA, ILS-TS, ILS-SA-TS, and MGA). However, the fitness value of the original KMA does not show any significant difference when compared to SA and ACS. On the other hand, despite excelling in terms of fitness value against most comparison algorithms, the original KMA again shows its weakness in time efficiency. Its running time is longer than 11 out of 14 comparison algorithms, but remains faster than ILS-SA, ILS-TS, and ILS-SA-TS. This pattern is consistent with the previous scenarios, confirming that running time is one of the main limitations of the original KMA.

The analysis continues to case 4, which is the last case in scenario 1. The main focus in this case is to prioritize the DOI rating value to evaluate the algorithm’s performance in optimizing the satisfaction level based on POI rating, without considering travel duration and cost. Table 8 presents the fitness value and running time of each algorithm, which serve as the basis for the Wilcoxon rank-sum test as shown in Table 9. From Table 9, it can be seen that the original KMA is able to compete in terms of fitness value with ACS, SA, DPSO, and TS, where no significant difference is found. On the other hand, the original KMA shows better fitness values than the majority of the comparison algorithms (KomoTrip, WOA-VNS, ACO, MOGA, BSO, ILS, ILS-SA, ILS-TS, ILS-SA-TS, and MGA). However, the original KMA records longer running time compared to most comparison algorithms, although it remains faster than ILS-TS and ILS-SA-TS, and shows no significant difference with ILS-SA. This pattern continues to be seen in every scenario in this experiment, so we can highlight that the main weakness of the original KMA lies in the aspect of time efficiency, even though this algorithm has an advantage in terms of fitness value.

**Table 8:** Fitness values and running times of the original KMA *vs*. other methods in case 4.

Method	Fitness value	Running time
**KomoTrip (Proposed method)**	0.7808	0.4078
Original KMA	0.7996	5.5286
ACS	0.7962	3.1692
SA	0.7934	0.9634
DPSO	0.7875	0.5405
TS	0.7860	0.6736
WOA-VNS	0.7826	0.3988
ACO	0.7757	2.4129
MOGA	0.7753	0.6611
BSO	0.7083	0.2269
ILS	0.7553	2.0342
ILS-SA	0.7685	6.5816
ILS-TS	0.7554	8.0981
ILS-SA-TS	0.7697	23.2847
MGA	0.7795	0.3393

**Table 9:** Statistical test results for the original KMA *vs*. other methods in case 4.

Comparative method	*p*-value	
	Fitness value	Running time
**KomoTrip (Proposed method)**	0.0054 (1)	6.9E−18 (−1)
ACS	0.5719 (0)	2.9E−08 (−1)
SA	0.3142 (0)	6.9E−18 (−1)
DPSO	0.1067 (0)	6.9E−18 (−1)
TS	0.0527 (0)	6.9E−18 (−1)
WOA-VNS	0.0155 (1)	6.9E−18 (−1)
ACO	0.0009 (1)	1.9E−16 (−1)
MOGA	0.0008 (1)	6.9E−18 (−1)
BSO	2.2E−14 (1)	6.9E−18 (−1)
ILS	3.4E−07 (1)	1.2E−15 (−1)
ILS-SA	5.2E−05 (1)	0.0519 (0)
ILS-TS	1.0E−07 (1)	1.9E−05 (1)
ILS-SA-TS	0.0001 (1)	3.9E−16 (1)
MGA	0.0020 (1)	6.9E−18 (−1)
Wilcoxon (0)	3	1
Wilcoxon (1)	11	2
Wilcoxon (−1)	0	11

Overall, the original KMA performs well in all four cases in this test scenario with various combinations of DOI values. It outperforms ACO, DPSO, TS, WOA-VNS, MOGA, BSO, ILS, ILS-SA, ILS-TS, ILS-SA-TS, MGA, and even KomoTrip in terms of fitness value, although not necessarily better than ACS and SA. However, the main drawback of the original KMA lies in its running time, which is consistently slower than the majority of comparison algorithms. This finding shows that while the original KMA excels in fitness value, its weakness in execution time opens up the opportunity for algorithms like KomoTrip to be a more efficient alternative. This is because KomoTrip has a much simpler process than the original KMA. Unlike the original KMA, KomoTrip is only performed in one optimization phase with a small population size (the population size is obtained through experimentation). In addition, KomoTrip does not require a sorting process for each generated solution because it is discrete from the beginning.

#### KomoTrip

After analyzing the performance of the original KMA and identifying its advantages and weaknesses, especially in terms of running time efficiency, the research focus turns to the main proposed algorithm, KomoTrip. In the first scenario, the settings remain the same, where we look at KomoTrip’s performance to accommodate the aggregation of all the demand criteria (assuming all are prioritized). This means that the DOI values for travel duration, cost, and rating are each set to 1. This indicates that the algorithm is designed to optimize multi-criteria simultaneously, with the goal of minimizing travel duration and cost, while maximizing satisfaction level based on POI rating. The fitness value and running time generated by KomoTrip, along with the other comparison algorithms in this scenario, are the same as summarized in Table 2. Meanwhile, the significance results based on the Wilcoxon rank-sum test are shown in Table 10, which gives an idea of the significance of the differences between the algorithm performances.

**Table 10:** Statistical test results for KomoTrip *vs*. other methods in case 1.

Comparative method	*p*-value	
	Fitness value	Running time
Original KMA	0.0011 (−1)	6.9E−18 (1)
ACS	0.0003 (−1)	6.9E−18 (1)
SA	0.0006 (−1)	1.0E−16 (1)
DPSO	0.1082 (0)	9.8E−05 (1)
WOA-VNS	0.8308 (0)	0.4863 (0)
TS	0.6741 (0)	7.0E−08 (1)
ACO	0.6842 (0)	6.9E−18 (1)
MOGA	0.0608 (0)	8.3E−07 (1)
BSO	1.1E−16 (1)	0.0709 (0)
ILS	5.8E−11 (1)	6.9E−18 (1)
ILS-SA	0.0007 (1)	6.9E−18 (1)
ILS-TS	0.0007 (1)	6.9E−18 (1)
ILS-SA-TS	3.2E−09 (1)	1.7E−17 (1)
MGA	0.0026 (−1)	0.0004 (1)
Wilcoxon (0)	5	2
Wilcoxon (1)	5	12
Wilcoxon (-1)	4	0

According to Table 10, KomoTrip shows superior fitness value over BSO, ILS, ILS-SA, ILS-TS, and ILS-SA-TS and is not significantly different from DPSO, WOA-VNS, TS, ACO, and MOGA. However, the fitness value of KomoTrip is still smaller than the original KMA, ACS, and SA. On the other hand, KomoTrip shows its main advantage in terms of running time, which is able to outperform the original KMA, ACS, SA, DPSO, TS, ACO, MOGA, ILS, ILS-SA, ILS-TS, ILS-SA-TS, and MGA. In addition, KomoTrip’s running time shows results that are not significantly different from WOA-VNS and BSO, making it a competitive alternative, especially when time efficiency is a top priority.

In case 2, the analysis focuses on the prioritization of the DOI travel duration value, excluding cost and rating. The resulting fitness and running time values in this case remain consistent as shown in Table 4, while Table 11 presents the Wilcoxon rank-sum test results for this case.

**Table 11:** Statistical test results for KomoTrip *vs*. other methods in case 2.

Comparative method	*p*-value	
	Fitness value	Running time
Original KMA	0.0013 (−1)	6.9E−18 (1)
ACS	0.0005 (−1)	6.9E−18 (1)
ACO	0.0019 (−1)	6.9E−18 (1)
SA	0.0024 (−1)	1.4E−16 (1)
DPSO	0.2059 (0)	8.2E−06 (1)
TS	0.3907 (0)	6.5E−08 (1)
WOA-VNS	0.4503 (0)	0.2730 (0)
MOGA	0.0544 (0)	6.5E−08 (1)
BSO	4.5E−11 (0)	0.0221 (−1)
ILS	1.7E−05 (1)	6.9E−18 (1)
ILS-SA	0.0097 (1)	6.9E−18 (1)
ILS-TS	0.0367 (1)	6.9E−18 (1)
ILS-SA-TS	8.4E−05 (1)	8.2E−18 (1)
MGA	0.1067 (0)	0.0018 (1)
Wilcoxon (0)	6	1
Wilcoxon (1)	4	12
Wilcoxon (−1)	4	1

Table 11 shows that KomoTrip produces a higher fitness value than BSO, ILS, ILS-SA, ILS-TS, ILS-SA-TS, and MGA. In addition, compared to DPSO, TS, WOA-VNS, and MOGA, the fitness value of KomoTrip is the same (not significantly different). However, the fitness value of KomoTrip is still lower than the original KMA, ACS, ACO, and SA. Another advantage of KomoTrip is seen in the speed of running time, where this method is faster than the original KMA, ACS, ACO, SA, DPSO, MOGA, ILS, ILS-SA, ILS-TS, ILS-SA-TS, and MGA. However, KomoTrip’s running time is still slower than BSO and does not show significant differences with TS and WOA-VNS.

The analysis then continues to case 3, which prioritizes the DOI cost value while travel duration and rating are not considered. In this case, the fitness value and running time for KomoTrip and several comparison algorithms remain the same as presented in Table 6. The Wilcoxon rank-sum test results to evaluate the significance of performance differences between algorithms in this scenario are shown in Table 12.

**Table 12:** Statistical test results for KomoTrip *vs*. other methods in case 3.

Comparative method	*p*-value	
	Fitness value	Running time
Original KMA	4.7E−06 (−1)	6.9E−18 (1)
SA	3.5E−06 (−1)	6.9E−18 (1)
ACS	6.3E−06 (−1)	6.9E−18 (1)
DPSO	0.0059 (−1)	0.0004 (1)
TS	0.2046 (0)	6.2E−09 (1)
WOA-VNS	0.4199 (0)	0.9725 (0)
MOGA	0.4081 (0)	3.4E−10 (1)
ACO	0.0463 (1)	6.9E−18 (1)
BSO	8.2E−18 (1)	2.4E−12 (−1)
ILS	7.4E−14 (1)	6.9E−18 (1)
ILS-SA	1.0E−07 (1)	6.9E−18 (1)
ILS-TS	0.0001 (1)	6.9E−18 (1)
ILS-SA-TS	6.3E−12 (1)	6.9E−18 (1)
MGA	0.0966 (0)	0.6541 (0)
Wilcoxon (0)	4	2
Wilcoxon (1)	6	11
Wilcoxon (−1)	4	1

Based on the results in Table 12, KomoTrip manages to excel in terms of fitness value compared to ACO, BSO, ILS, ILS-SA, ILS-TS, and ILS-SA-TS. Meanwhile, comparison with TS, WOA-VNS, MOGA, and MGA shows that KomoTrip’s performance on these metrics is not significantly different. However, when compared to the original KMA, SA, ACS, and DPSO, KomoTrip still shows smaller results in terms of fitness value. In terms of running time, KomoTrip is able to show the higher efficiency than the original KMA, SA, ACS, DPSO, TS, MOGA, ACO, ILS, ILS-SA, ILS-TS, and ILS-SA-TS. However, the execution time of this algorithm is still slower than BSO and not significantly different from WOA-VNS and MGA.

In the last case, the DOI rating value is prioritized, while travel duration and cost are ignored. The results of both metrics (fitness value and running time) achieved in this case remain consistent with those shown in Table 8, while the Wilcoxon rank-sum test results are presented in Table 13.

**Table 13:** Statistical test results for KomoTrip *vs*. other methods in case 4.

Comparative method	*p*-value	
	Fitness value	Running time
Original KMA	0.0054 (−1)	6.9E−18 (1)
ACS	0.0202 (−1)	6.9E−18 (1)
SA	0.0502 (0)	1.3E−16 (1)
DPSO	0.2096 (0)	8.0E−05 (1)
TS	0.3926 (0)	2.0E−12 (1)
WOA-VNS	0.7854 (0)	0.5037 (0)
ACO	0.4949 (0)	6.9E−18 (1)
MOGA	0.4421 (0)	5.4E−08 (1)
BSO	7.6E−12 (1)	1.5E−08 (−1)
ILS	0.1477 (0)	6.9E−18 (1)
ILS-SA	0.0642 (0)	6.9E−18 (1)
ILS-TS	0.0008 (1)	6.9E−18 (1)
ILS-SA-TS	0.0013 (1)	2.9E−17 (1)
MGA	0.6691 (0)	0.2198 (0)
Wilcoxon (0)	9	2
Wilcoxon (1)	3	11
Wilcoxon (−1)	2	1

From Table 13, it can be seen that the fitness value of KomoTrip is consistently superior to BSO, ILS-TS, and ILS-SA-TS in all cases in this experiment. However, KomoTrip’s fitness value still lags behind the original KMA and ACS. Meanwhile, for SA, DPSO, TS, WOA-VNS, ACO, MOGA, ILS, ILS-SA, and MGA, there is no significant difference in fitness value when compared to KomoTrip. In terms of running time, KomoTrip shows a much better speed than the original KMA, ACS, SA, DPSO, TS, ACO, MOGA, ILS, ILS-SA, ILS-TS, and ILS-SA-TS. However, KomoTrip’s running time is not significantly different from WOA-VNS and MGA, and is still slower than BSO.

Overall, the results of general performance testing focused on KomoTrip, KomoTrip shows competitive performance even though its fitness value is not always the highest (in major cases are not significant different with others). In each case, KomoTrip’s fitness value is on average lower than the original KMA, ACS, and SA. However, the main advantage of KomoTrip lies in its running time efficiency, which is faster than most of the other algorithms (original KMA, ACS, ACO, MOGA, DPSO, SA, TS, ILS, ILS-SA, ILS-TS, and ILS-SA-TS). This time efficiency is a key point, considering that multi-day travel route problems require algorithms that can provide recommendation results quickly.

### Scenario 2: DOI combination test

This experiment is conducted by giving various combinations of DOI values to the original KMA, KomoTrip, and other methods. The purpose of this experiment is to simulate real-life conditions, where users can specify DOI values according to their preferences. In other words, this test aims to evaluate the performance of each method, especially the original KMA and KomoTrip, based on varying preferences. Within each DOI combination (there are 10 random combinations in total, as shown in Table 14), the experiment is conducted for 50 repetitions, with each trial involving 30 POIs selected for a three-day trip.

**Table 14:** DOI values for the 10 random combinations.

Combination	DOI		
	Travel duration	Cost	Rating
1	0.15	0.45	0.77
2	0.16	0.65	0.12
3	0.19	0.35	0.63
4	0.46	0.05	0.93
5	0.56	0.9	0.82
6	0.71	0.73	0.43
7	0.77	0.2	0.18
8	0.8	0.53	0.08
9	0.95	0.34	0.31
10	0.96	0.21	0.96

#### Original KMA

As the basic algorithm used in this experiment, the original KMA is evaluated to determine its performance on various combinations of DOIs. With 10 combinations of DOI values tested, the results of the original KMA can be used as a reference to compare the performance of the proposed method, KomoTrip. The fitness value results achieved by the original KMA for each DOI combination are summarized in Table 15, which forms the basis for further evaluation through the Wilcoxon rank-sum test. Table 16 provides the test results to assess whether the difference in fitness values between the original KMA and the other comparison algorithms is significant.

**Table 15:** Fitness value comparison of the original KMA and other methods for different DOI combinations.

Method	Combination									
	1	2	3	4	5	6	7	8	9	10
**KomoTrip (Proposed method)**	0.7518	0.7695	0.7468	0.7035	0.6910	0.6759	0.6668	0.6665	0.6392	0.6295
Original KMA	0.7633	0.7859	0.7636	0.7168	0.7058	0.6892	0.6801	0.6796	0.6537	0.6455
ACO	0.7467	0.7637	0.7438	0.7027	0.6888	0.6714	0.6640	0.6665	0.6370	0.6332
ACS	0.7640	0.7878	0.7636	0.7163	0.7080	0.6918	0.6816	0.6809	0.6536	0.6460
BSO	0.6726	0.6716	0.6769	0.6511	0.5980	0.5900	0.6076	0.5879	0.5775	0.5821
MOGA	0.7416	0.7591	0.7394	0.6959	0.6812	0.6645	0.6567	0.6517	0.6273	0.6248
DPSO	0.7549	0.7784	0.7538	0.7074	0.6984	0.6809	0.6715	0.6714	0.6431	0.6358
SA	0.7630	0.7876	0.7612	0.7130	0.7078	0.6911	0.6793	0.6799	0.6517	0.6435
TS	0.7504	0.7706	0.7482	0.7018	0.6911	0.6750	0.6639	0.6641	0.6363	0.6313
WOA-VNS	0.7473	0.7685	0.7469	0.7013	0.6895	0.6725	0.6616	0.6633	0.6363	0.6287
ILS	0.7112	0.7161	0.7136	0.6798	0.6385	0.6274	0.6364	0.6231	0.6076	0.6067
ILS-SA	0.7273	0.7420	0.7281	0.6890	0.6602	0.6418	0.6492	0.6407	0.6196	0.6168
ILS-TS	0.7103	0.7146	0.7136	0.6795	0.6377	0.6239	0.6347	0.6198	0.6036	0.6059
ILS-SA-TS	0.7282	0.7441	0.7299	0.6919	0.6625	0.6478	0.6495	0.6386	0.6227	0.6187
MGA	0.7509	0.7771	0.7525	0.7044	0.7048	0.6868	0.6724	0.6788	0.6511	0.6437

**Table 16:** Statistical analysis of the original KMA *vs*. other methods based on fitness values using the Wilcoxon rank-sum test for different DOI combinations.

Method	Combination										Wilcoxon		
	1	2	3	4	5	6	7	8	9	10	0	1	−1
**KomoTrip (Proposed method)**	0.0332 (1)	0.0013 (1)	0.0017 (1)	0.0229 (1)	0.0105 (1)	0.0072 (1)	0.0019 (1)	0.0061 (1)	0.0005 (1)	0.0038 (1)	0	10	0
ACO	0.0009 (1)	1.1E−05 (1)	0.0002 (1)	0.0133 (1)	0.0018 (1)	0.0003 (1)	0.0001 (1)	0.0022 (1)	0.0001 (1)	0.0191 (1)	0	10	0
ACS	0.9615 (0)	0.6148 (0)	0.9780 (0)	0.9670 (0)	0.5259 (0)	0.4120 (0)	0.7880 (0)	0.8254 (0)	0.8577 (0)	0.7880 (0)	10	0	0
BSO	5.8E−16 (1)	7.7E−18 (1)	1.5E−15 (1)	1.3E−11 (1)	1.0E−17 (1)	7.3E−18 (1)	3.5E−16 (1)	1.3E−17 (1)	7.8E−17 (1)	4.3E−13 (1)	0	10	0
MOGA	0.0002 (1)	4.2E−07 (1)	6.1E−06 (1)	0.0006 (1)	1.8E−05 (1)	4.7E−07 (1)	3.0E−07 (1)	3.9E−09 (1)	3.1E−09 (1)	0.0002 (1)	0	10	0
DPSO	0.0688 (0)	0.0571 (0)	0.0171 (1)	0.0709 (0)	0.1037 (0)	0.0274 (1)	0.0139 (1)	0.0147 (1)	0.0038 (1)	0.0386 (1)	4	6	0
SA	0.8200 (0)	0.7097 (0)	0.7827 (0)	0.4649 (0)	0.6003 (0)	0.4993 (0)	0.7827 (0)	0.9670 (0)	0.4649 (0)	0.7251 (0)	10	0	0
TS	0.0187 (1)	0.0013 (1)	0.0019 (1)	0.0058 (1)	0.0027 (1)	0.0010 (1)	6.8E−05 (1)	0.0001 (1)	2.4E−05 (1)	0.0038 (1)	0	10	0
WOA-VNS	0.0032 (1)	0.0005 (1)	0.0011 (1)	0.0080 (1)	0.0017 (1)	0.0001 (1)	1.5E−05 (1)	4.8E−05 (1)	4.8E−05 (1)	0.0022 (1)	0	10	0
ILS	1.8E−10 (1)	9.2E−14 (1)	2.2E−11 (1)	5.0E−07 (1)	2.6E−12 (1)	1.6E−14 (1)	1.6E−11 (1)	8.3E−14 (1)	5.6E−13 (1)	4.1E−09 (1)	0	10	0
ILS-SA	1.7E−06 (1)	9.7E−09 (1)	4.3E−07 (1)	2.7E−05 (1)	2.4E−09 (1)	1.4E−11 (1)	7.0E−09 (1)	6.6E−11 (1)	1.8E−09 (1)	6.3E−06 (1)	0	10	0
ILS-TS	7.3E−11 (-1)	2.2E−14 (1)	8.4E−11 (1)	2.0E−07 (1)	1.0E−13 (1)	3.0E−14 (1)	1.1E−11 (1)	1.7E−14 (1)	4.5E−13 (1)	4.4E−09 (1)	0	10	0
ILS-SA-TS	4.3E−07 (1)	9.3E−09 (1)	9.2E−07 (1)	0.0001 (1)	1.3E−09 (1)	2.0E−11 (1)	1.9E−08 (1)	1.3E−10 (1)	8.6E−09 (1)	8.5E−06 (1)	0	10	0
MGA	0.0202 (0)	0.0823 (0)	0.0184 (1)	0.0251 (1)	0.9890 (0)	0.6641 (0)	0.0471 (1)	0.8040 (0)	0.4524 (0)	0.7512 (0)	7	3	0

Based on Table 16, original KMA consistently shows its superiority in terms of fitness value over KomoTrip, ACO, BSO, MOGA, TS, WOA-VNS, ILS, ILS-SA, ILS-TS, and ILS-SA-TS across all DOI combinations tested. Against DPSO, the original KMA performs better in 6 out of 10 combinations, while the remaining four show no statistically significant difference. In addition, the performance of the original KMA is not significantly different when compared to ACS and SA, showing relatively equal fitness value results among the two. Meanwhile, for MGA, 7 out of 10 combinations show no significant difference, while the remaining three are statistically significant. However, the original KMA’s advantage in fitness value is not matched by its efficiency in terms of running time. The Wilcoxon rank-sum test on Table 17, which is based on the running time data from Table 18, shows that the original KMA has a significantly slower execution time than 11 out of 14 comparison algorithms (KomoTrip, ACO, ACS, BSO, MOGA, DPSO, SA, TS, WOA-VNS, ILS, and MGA). This consistency is seen in all DOI combinations tested, confirming that running time is the main drawback of this algorithm, although it consistently outperforms ILS-SA, ILS-TS, and ILS-SA-TS in this aspect. The adaptation of the original KMA to solve discrete problems causes the algorithm to have a higher complexity than when using the original KMA on continuous problems. This is due to the need to sort each solution (Komodo) generated by the original KMA to get the final solution and calculate its fitness.

**Table 17:** Running time comparison of the original KMA and other methods for different DOI combinations.

Method	Combination			
	1	2	3	4	5	6	7	8	9	10
**KomoTrip (Proposed method)**	0.4977	0.4427	0.4490	0.4921	0.4552	0.4869	0.4920	0.4990	0.4473	0.4027
Original KMA	5.0105	5.0750	6.0172	5.9040	5.2323	4.4898	5.6721	5.3675	5.6924	5.4606
ACO	3.1534	2.9193	2.7594	2.8419	2.8356	2.9968	3.0073	3.0249	2.9636	2.8276
ACS	4.3205	4.5932	4.2764	4.1778	4.1788	4.5442	4.3002	4.8338	4.5959	4.2048
BSO	0.4013	0.3432	0.3699	0.3931	0.3756	0.3783	0.3670	0.3680	0.3618	0.3701
MOGA	0.7384	0.6458	0.6834	0.7398	0.7139	0.6635	0.7282	0.6641	0.7111	0.6879
DPSO	0.6109	0.6577	0.6747	0.6954	0.6242	0.6434	0.6964	0.6851	0.6220	0.6637
SA	1.0035	0.9438	0.9457	0.9753	0.9427	0.9690	0.9502	0.9693	0.9613	0.9427
TS	0.6840	0.6569	0.6566	0.6753	0.6604	0.6703	0.6622	0.6732	0.6644	0.6537
WOA-VNS	0.4150	0.4368	0.4495	0.4277	0.4427	0.4299	0.4045	0.4164	0.4327	0.4432
ILS	2.6629	2.5989	2.6948	2.5889	2.3688	2.7089	2.7409	2.6906	2.7186	2.3919
ILS-SA	7.2745	8.1924	8.1737	7.4676	8.2611	6.7670	8.1035	7.7690	7.4426	7.7368
ILS-TS	10.4887	10.9529	10.2706	10.5353	10.5986	10.6565	10.3365	11.2222	9.9295	10.2047
ILS-SA-TS	21.3252	21.5509	22.0416	19.3625	22.2947	21.0159	20.0678	22.0658	21.9869	20.2193
MGA	0.5370	0.5286	0.5375	0.5167	0.5544	0.5473	0.5285	0.5460	0.5569	0.5537

**Table 18:** Statistical analysis of the original KMA *vs*. other methods based on running time using the Wilcoxon rank-sum test for different DOI combinations.

Method	Combination										Wilcoxon		
	1	2	3	4	5	6	7	8	9	10	0	1	−1
**KomoTrip (Proposed method)**	6.9E−18 (−1)	6.9E−18 (−1)	6.9E−18 (−1)	6.9E−18 (−1)	6.9E−18 (−1)	6.9E−18 (−1)	6.9E−18 (−1)	6.9E−18 (−1)	6.9E−18 (−1)	6.9E−18 (−1)	0	0	10
ACO	4.5E−05 (−1)	8.3E−09 (−1)	7.9E−13 (−1)	1.4E−11 (−1)	1.9E−07 (−1)	0.0004 (−1)	1.2E−10 (−1)	7.8E−10 (−1)	4.4E−11 (−1)	1.5E−13 (−1)	0	0	10
ACS	0.3076 (−1)	0.2303 (−1)	2.7E−05 (-1)	0.0002 (−1)	0.0811 (−1)	0.6944 (−1)	0.0051 (−1)	0.1723 (−1)	0.0083 (−1)	0.0006 (−1)	0	0	10
BSO	6.9E−18 (−1)	6.9E−18 (−1)	6.9E−18 (−1)	6.9E−18 (−1)	6.9E−18 (−1)	6.9E−18 (−1)	6.9E−18 (−1)	6.9E−18 (−1)	6.9E−18 (−1)	6.9E−18 (−1)	0	0	10
MOGA	6.9E−18 (−1)	6.9E−18 (−1)	6.9E−18 (−1)	6.9E−18 (−1)	6.9E−18 (−1)	6.9E−18 (−1)	6.9E−18 (−1)	6.9E−18 (−1)	6.9E−18 (−1)	6.9E−18 (−1)	0	0	10
DPSO	6.9E−18 (−1)	6.9E−18 (−1)	6.9E−18 (−1)	6.9E−18 (−1)	6.9E−18 (−1)	6.9E−18 (−1)	6.9E−18 (−1)	6.9E−18 (−1)	6.9E−18 (−1)	6.9E−18 (−1)	0	0	10
SA	6.9E−18 (−1)	6.9E−18 (−1)	6.9E−18 (−1)	6.9E−18 (−1)	6.9E−18 (−1)	6.9E−18 (−1)	6.9E−18 (−1)	6.9E−18 (−1)	6.9E−18 (−1)	6.9E−18 (−1)	0	0	10
TS	6.9E−18 (−1)	6.9E−18 (−1)	6.9E−18 (−1)	6.9E−18 (−1)	6.9E−18 (−1)	6.9E−18 (−1)	6.9E−18 (−1)	6.9E−18 (−1)	6.9E−18 (−1)	6.9E−18 (−1)	0	0	10
WOA-VNS	6.9E−18 (−1)	6.9E−18 (−1)	6.9E−18 (−1)	6.9E−18 (−1)	6.9E−18 (−1)	6.9E−18 (−1)	6.9E−18 (−1)	6.9E−18 (−1)	6.9E−18 (−1)	6.9E−18 (−1)	0	0	10
ILS	2.1E−07 (−1)	1.1E−09 (−1)	6.5E−13 (−1)	1.3E−12 (−1)	1.5E−10 (−1)	7.7E−07 (−1)	8.8E−11 (−1)	2.9E−12 (−1)	7.3E−12 (−1)	9.7E−14 (−1)	0	0	10
ILS-SA	0.0002 (1)	4.1E−06 (1)	0.0027 (1)	0.0251 (1)	5.5E−06 (1)	2.3E−05 (1)	0.0003 (1)	6.4E−05 (1)	0.0049 (1)	0.0002 (1)	0	10	0
ILS-TS	3.8E−11 (1)	1.1E−10 (1)	7.9E−08 (1)	9.5E−07 (1)	2.5E−09 (1)	4.6E−11 (1)	1.3E−08 (1)	1.0E−10 (1)	6.0E−08 (1)	1.8E−09 (1)	0	10	0
ILS-SA-TS	2.9E−17 (1)	1.4E−17 (1)	5.5E−17 (1)	7.8E−17 (1)	6.5E−17 (1)	2.7E−17 (1)	7.3E−16 (1)	1.5E−17 (1)	2.4E−17 (1)	8.2E−18 (1)	0	10	0
MGA	6.9E−18 (−1)	6.9E−18 (−1)	6.9E−18 (−1)	6.9E−18 (−1)	6.9E−18 (−1)	6.9E−18 (−1)	6.9E−18 (−1)	6.9E−18 (−1)	6.9E−18 (−1)	6.9E−18 (−1)	0	0	10

The results of the DOI combinations test are in line with the findings in scenario 1. In both tests, the original KMA consistently shows an advantage in fitness value over most comparison algorithms. In Scenario 1, it outperforms KomoTrip, ACO, DPSO, TS, WOA-VNS, MOGA, BSO, ILS, ILS-SA, ILS-TS, ILS-SA-TS, and MGA. A similar pattern is observed in the current scenario, where the original KMA maintains its advantage over KomoTrip, ACO, BSO, MOGA, TS, WOA-VNS, ILS, ILS-SA, ILS-TS, and ILS-SA-TS. The running time of the original KMA, which is consistently slower than most comparison algorithms, remains a major challenge in both scenarios. This finding indicates that although the original KMA excels in solution quality, time efficiency remains an aspect that needs to be improved for practical applications.

#### KomoTrip

After the previous test results show the weakness of the original KMA in terms of running time, the focus of analysis shifts to KomoTrip as the proposed method in this study. Designed as a development of the original KMA, KomoTrip is tested on the same 10 DOI combinations to evaluate its efficiency in running time as well as its performance in generating fitness values. This test aims to assess the extent to which KomoTrip is able to compete with other algorithms and provide more optimal results. The fitness values generated by KomoTrip in each DOI combination are summarized in Table 15, while the Wilcoxon rank-sum test results to evaluate the significance of the performance difference are shown in Table 19.

**Table 19:** Statistical analysis of KomoTrip *vs*. other methods based on fitness values using the Wilcoxon rank-sum test for different DOI combinations.

Method	Combination										Wilcoxon		
	1	2	3	4	5	6	7	8	9	10	0	1	−1
**KomoTrip (Proposed method)**	0.0332 (−1)	0.0013 (−1)	0.0017 (−1)	0.0229 (−1)	0.0105 (−1)	0.0072 (−1)	0.0019 (−1)	0.0061 (−1)	0.0005 (−1)	0.0038 (−1)	0	0	10
ACO	0.1926 (0)	0.1811 (0)	0.6100 (0)	0.9670 (0)	0.7669 (0)	0.4401 (0)	0.4319 (0)	0.9231 (0)	0.6491 (0)	0.4524 (0)	10	0	0
ACS	0.0320 (−1)	0.0003 (−1)	0.0021 (−1)	0.0420 (−1)	0.0030 (−1)	0.0013 (−1)	0.0006 (−1)	0.0028 (−1)	0.0004 (−1)	0.0021 (−1)	0	0	10
BSO	7.1E−14 (1)	9.8E−17 (1)	1.4E−12 (1)	3.1E−09 (1)	2.1E−16 (1)	4.4E−16 (1)	3.3E−13 (1)	3.5E−16 (1)	1.4E−14 (1)	3.1E−09 (1)	0	10	0
MOGA	0.0361 (1)	0.0299 (1)	0.1128 (0)	0.1879 (0)	0.1052 (0)	0.0202 (1)	0.0112 (1)	0.0008 (1)	0.0030 (1)	0.2670 (0)	4	4	2
DPSO	0.9396 (0)	0.0873 (0)	0.2582 (0)	0.5487 (0)	0.2582 (0)	0.4524 (0)	0.4159 (0)	0.3310 (0)	0.3175 (0)	0.3379 (0)	10	0	0
SA	0.0580 (0)	0.0003 (−1)	0.0041 (−1)	0.0994 (0)	0.0035 (−1)	0.0011 (−1)	0.0056 (−1)	0.0045 (−1)	0.0018 (−1)	0.0067 (−1)	2	0	8
TS	0.7303 (0)	0.8795 (0)	0.6995 (0)	0.9176 (0)	0.9725 (0)	0.8040 (0)	0.3345 (0)	0.4607 (0)	0.4906 (0)	0.6691 (0)	10	0	0
WOA-VNS	0.3276 (0)	0.8469 (0)	0.9341 (0)	0.8632 (0)	0.7669 (0)	0.3665 (0)	0.1383 (0)	0.4319 (0)	0.4734 (0)	0.7355 (0)	10	0	0
ILS	8.2E−08 (1)	6.3E−10 (1)	1.1E−06 (1)	0.0004 (1)	5.0E−09 (1)	1.8E−10 (1)	2.4E−07 (1)	1.4E−09 (1)	1.9E−08 (1)	0.0001 (1)	0	10	0
ILS-SA	0.0003 (1)	6.2E−05 (1)	0.0028 (1)	0.0131 (1)	1.9E−05 (1)	2.9E−07 (1)	0.0002 (1)	5.0E−06 (1)	0.0001 (1)	0.0315 (1)	0	10	0
ILS-TS	6.0E−08 (1)	8.4E−11 (1)	3.4E−06 (1)	0.0005 (1)	2.0E−10 (1)	1.2E−10 (1)	1.3E−07 (1)	1.5E−10 (1)	7.0E−09 (1)	9.5E−05 (1)	0	10	0
ILS-SA-TS	0.0003 (1)	0.0001 (1)	0.0067 (1)	0.0776 (1)	3.7E−05 (1)	2.4E−06 (1)	0.0004 (1)	6.5E−06 (1)	0.0009 (1)	0.0361 (1)	0	10	0
MGA	0.6541 (0)	0.1659 (0)	0.3276 (0)	0.7933 (0)	0.0150 (−1)	0.0221 (−1)	0.1879 (0)	0.0081 (−1)	0.0048 (−1)	0.0083 (−1)	5	0	5

The evaluation results in Table 19 show that the fitness value of KomoTrip consistently outperforms BSO, ILS, ILS-SA, ILS-TS, and ILS-SA-TS in all tested DOI combinations, and the results show no significant difference with ACO, DPSO, TS, and WOA-VNS. On the other hand, KomoTrip’s fitness value is still lower than the original KMA and ACS in all combinations, and loses to SA and MGA in eight and five out of 10 combinations, respectively. Meanwhile, in comparison with MOGA, KomoTrip excels in six combinations, while the remaining four show no significant difference.

Although KomoTrip’s fitness value is not able to surpass the original KMA, ACS, and SA in most of the DOI combinations, this algorithm shows a significant advantage in terms of running time. Information related to the execution time of KomoTrip and other comparison algorithms is summarized in Table 17, which is the basis for the Wilcoxon rank-sum test in Table 20. Based on Table 20, KomoTrip records faster execution times than the original KMA, ACO, ACS, MOGA, DPSO, SA, TS, ILS, ILS-SA, ILS-TS, ILS-SA-TS, and MGA across all DOI combinations. For WOA-VNS, KomoTrip’s running time in 9 out of 10 combinations shows no significant difference. In contrast, compared to BSO, KomoTrip’s running time is slower in six out of 10 combinations, although no significant difference is found in four of them.

**Table 20:** Statistical analysis of KomoTrip *vs*. other methods based on running time using the Wilcoxon rank-sum test for different DOI combinations.

Method	Combination										Wilcoxon		
	1	2	3	4	5	6	7	8	9	10	0	1	−1
**KomoTrip (Proposed method)**	6.9E−18 (1)	6.9E−18 (1)	6.9E−18 (1)	6.9E−18 (1)	6.9E−18 (1)	6.9E−18 (1)	6.9E−18 (1)	6.9E−18 (1)	6.9E−18 (1)	6.9E−18 (1)	0	10	0
ACO	6.9E−18 (1)	6.9E−18 (1)	6.9E−18 (1)	6.9E−18 (1)	6.9E−18 (1)	6.9E−18 (1)	6.9E−18 (1)	6.9E−18 (1)	6.9E−18 (1)	6.9E−18 (1)	0	10	0
ACS	6.9E−18 (1)	6.9E−18 (1)	6.9E−18 (1)	6.9E−18 (1)	6.9E−18 (1)	6.9E−18 (1)	6.9E−18 (1)	6.9E−18 (1)	6.9E−18 (1)	6.9E−18 (1)	0	10	0
BSO	0.1659 (0)	0.0076 (−1)	0.0657 (0)	0.0304 (−1)	0.0848 (0)	0.0047 (−1)	0.0373 (−1)	0.0008 (−1)	0.0126 (−1)	0.4734 (0)	4	0	6
MOGA	1.7E−05 (1)	1.1E−05 (1)	2.3E−06 (1)	1.2E−05 (1)	2.9E−06 (1)	6.8E−04 (1)	2.4E−05 (1)	6.4E−04 (1)	3.0E−08 (1)	2.6E−09 (1)	0	10	0
DPSO	1.5E−03 (1)	2.2E−07 (1)	2.3E−07 (1)	6.1E−06 (1)	2.7E−05 (1)	2.3E−04 (1)	7.2E−05 (1)	3.5E−05 (1)	1.2E−05 (1)	2.2E−09 (1)	0	10	0
SA	1.6E−13 (1)	1.3E−16 (1)	6.9E−18 (1)	1.7E−16 (1)	1.2E−16 (1)	6.9E−18 (1)	4.1E−12 (1)	6.9E−18 (1)	6.9E−18 (1)	6.9E−18 (1)	0	10	0
TS	1.5E−05 (1)	2.8E−10 (1)	1.1E−08 (1)	5.4E−06 (1)	2.3E−07 (1)	2.0E−06 (1)	7.2E−06 (1)	1.0E−06 (1)	8.7E−14 (1)	2.5E−11 (1)	0	10	0
WOA-VNS	0.6417 (0)	0.9122 (0)	0.5813 (0)	0.5037 (0)	0.6051 (0)	0.2884 (0)	0.6541 (0)	0.0274 (−1)	0.6245 (0)	0.0647 (0)	9	0	1
ILS	2.9E−17 (1)	1.0E−17 (1)	6.9E−18 (1)	8.2E−18 (1)	8.2E−18 (1)	8.7E−18 (1)	3.9E−17 (1)	2.1E−17 (1)	6.9E−18 (1)	6.9E−18 (1)	0	10	0
ILS-SA	6.9E−18 (1)	6.9E−18 (1)	6.9E−18 (1)	6.9E−18 (1)	6.9E−18 (1)	6.9E−18 (1)	6.9E−18 (1)	6.9E−18 (1)	6.9E−18 (1)	6.9E−18 (1)	0	10	0
ILS-TS	6.9E−18 (1)	6.9E−18 (1)	6.9E−18 (1)	6.9E−18 (1)	6.9E−18 (1)	6.9E−18 (1)	6.9E−18 (1)	6.9E−18 (1)	6.9E−18 (1)	6.9E−18 (1)	0	10	0
ILS-SA-TS	6.9E−18 (1)	6.9E−18 (1)	6.9E−18 (1)	6.9E−18 (1)	6.9E−18 (1)	6.9E−18 (1)	6.9E−18 (1)	6.9E−18 (1)	6.9E−18 (1)	6.9E−18 (1)	0	10	0
MGA	0.0133 (1)	0.0024 (1)	0.0028 (1)	0.1723 (1)	0.0011 (1)	0.0637 (1)	0.0647 (1)	0.0269 (1)	0.0022 (1)	3.6E−07 (1)	0	10	0

Evaluation of our proposed method, KomoTrip, reveals a combination of strengths and weaknesses. In both scenario 1 and scenario 2, KomoTrip’s fitness value is consistently lower than the original KMA, ACS, and SA. However, this weakness in terms of fitness value is offset by a significant advantage in running time. This algorithm has better execution time than the majority of algorithms. This efficiency is an important plus for KomoTrip, as it is able to offer faster solutions without completely compromising the quality of the solution.

### Scenario 3: various numbers of POIs test

**Figure 18:**
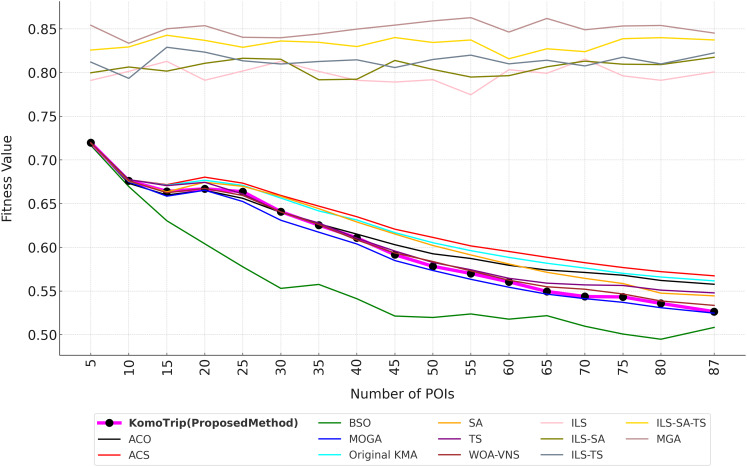
Fitness value trends across different numbers of POIs.

**Figure 19:**
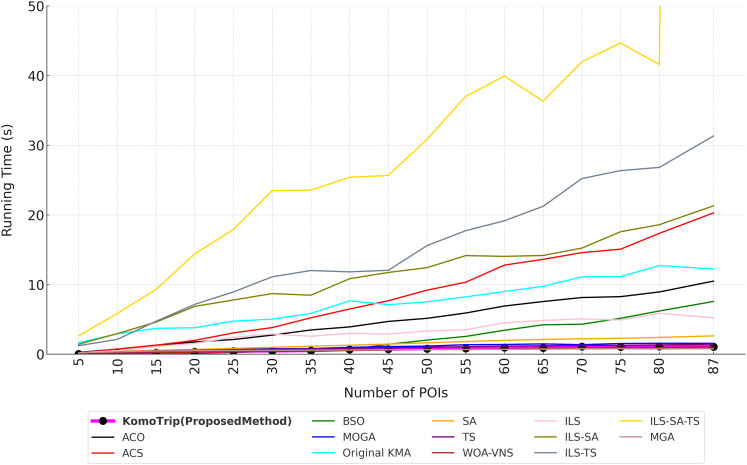
Running time trends across different numbers of POIs.

This experiment is designed to optimize travel routes with varying numbers of POIs, ranging from five to 87 POIs. For each number of POIs, 50 trials are conducted to ensure consistent results. All DOI values are set to 1, while the travel duration is set for 3 days. The main objective of this experiment is to analyze the trend of fitness value and running time based on changes in the number of POIs that represent the user’s desired POIs. In addition, this experiment also aims to evaluate the robustness of the algorithm to changes in problem dimension and size through variations in desired POI. Figure 18 depicts the trend of fitness value, which shows the change of algorithm solution quality as the number of POIs increases, while Fig. 19 presents the trend of running time, which reflects the efficiency of algorithm execution time at different problem scales. These two graphs provide a comprehensive overview of the algorithm’s performance in terms of solution quality and running time speed on various problem sizes.

From the analysis in Fig. 18, it is clear that the trend of fitness value for KomoTrip, ACO, ACS, MOGA, original KMA, DPSO, SA, TS, and WOA-VNS tends to stabilize after the number of POIs reaches 20. In contrast, BSO shows a more volatile pattern, especially after the number of POIs exceeds 30. A similar stable trend is also observed in ILS, ILS-SA, ILS-TS, and ILS-SA-TS across all POI counts. Meanwhile, MGA maintains a consistently high trend throughout the test range. In terms of running time, as shown in Fig. 19, KomoTrip, ACO, MOGA, DPSO, SA, TS, and WOA-VNS show efficient and consistent performance for various numbers of POIs. KomoTrip has the fastest running time, as seen from its position at the bottom of the graph compared to ACO, ACS, MOGA, original KMA, DPSO, SA, and TS. Although at POI counts below 45, KomoTrip has a running time comparable to BSO, at larger POI counts (starting from 45 POIs), KomoTrip consistently outperforms BSO in terms of execution speed. On the other hand, ILS, ILS-SA, ILS-TS, and ILS-SA-TS exhibit higher execution times, with ILS-SA-TS being the slowest overall, especially as the number of POIs increases.

Overall, it can be concluded that KomoTrip is robust in terms of both fitness value and running time. On the other hand, while the original KMA excels in terms of fitness value, it has the disadvantage of less efficient running time and tends to fluctuate, making it less ideal for applications that require high time efficiency.

### Model benchmarking using public dataset

To analyze the performance of KomoTrip against some TOPTW heuiristics, we also compare the results of fitness value through Solomon dataset (Solomon, 1987) (https://www.mech.kuleuven.be/en/cib/op). In this study, we employ the Multi-Constraint Team Orienteering Problem with (Multiple) Time Windows (MCTOPMTW) instances in Solomon dataset, as it provides the closest approximation to the problem addressed by KomoTrip. This dataset considers a single criteria of user need, *i.e*., budget minimization, without incorporating the Degree of Interest (*i.e*., user-specific preference weights across different criteria). Nevertheless, given the single-criteria formulation and the presence of time window constraints within the Solomon dataset, it remains suitable for assessing the relative performance of KomoTrip against existing methods.

In the Solomon dataset, the instances cxxx, rxxx, rcxxx represent clustered nodes, random nodes and random-clustered nodes, respectively. For evaluation, KomoTrip was executed for each instance through 30 trials. The comparison algorithms were ILS, ILS-SA, ILS-TS, and ILS-SA-TS, which are three high-performance TOPTW heuristics that have been executed on MCTOPTW instances in the Solomon Dataset (Sylejmani et al., 2024). Tables 21 to 24 show the profits generated by KomoTrip, along with the gap values generated by the five algorithms, for each $m = 1 - 4$. The gap is calculated by referring to the proportion of the difference between the Best Known Solution (https://www.mech.kuleuven.be/en/cib/op) and the profits generated by each algorithm. The profit values from the benchmarking algorithms refer to Sylejmani et al. (2024). In Tables 21 to 24, we display the gap (%) of the profits generated by each of these algorithms against the *best known solution* (BK). The best result from each dataset, highlighted in bold.

**Table 21:** Results on Solomon benchmark with 1 day (*m* = 1 tour). Bold values denote the best results; negative values indicate the results exceeding the BK.

Instance	BK	KomoTrip profit		Gap (%)				
		Best	Average	KomoTrip (Proposed method)	ILS	ILS-SA	ILS-TS	ILS-SA-TS
1-c101	320	281	270.69	**12.19**	12.50	12.50	12.50	12.50
1-c102	360	348	349.17	3.33	**2.78**	**2.78**	2.78	2.78
1-c103	400	382	379.49	4.50	5.00	5.00	5.00	**2.50**
1-c104	420	413	408.63	**1.67**	2.38	2.38	2.38	2.38
1-c105	340	343	319.89	**−0.88**	0.00	0.00	0.00	0.00
1-c106	340	337	338.34	0.88	**0.00**	**0.00**	**0.00**	**0.00**
1-c107	370	363	355.26	1.89	**0.00**	**0.00**	**0.00**	**0.00**
1-c108	370	325	314.03	12.16	**10.81**	**10.81**	**10.81**	**10.81**
1-c109	380	374	366.37	1.58	**0.00**	**0.00**	**2.63**	**0.00**
1-r101	198	159	143.6	19.70	**18.69**	**18.69**	**18.69**	**18.69**
1-r102	286	277	273.38	**3.15**	**3.15**	**3.15**	**3.15**	**3.15**
1-r103	293	292	291.02	0.34	**0.00**	**0.00**	**0.00**	**0.00**
1-r104	303	259	245.17	14.52	13.20	**8.91**	12.87	8.91
1-r105	247	239	226.8	3.24	**2.83**	**2.83**	**2.83**	**2.83**
1-r106	293	287	284.96	**2.05**	2.39	2.39	2.39	2.39
1-r107	299	288	271.94	**3.68**	4.01	4.01	4.01	4.01
1-r108	308	296	295.06	3.90	3.57	3.57	6.17	**3.57**
1-r109	277	249	243.3	10.11	9.39	9.39	**3.25**	9.39
1-r110	284	270	266.92	4.93	**3.52**	**3.52**	**3.52**	3.52
1-r111	297	297	277.59	0.00	1.68	1.68	1.68	**0.00**
1-r112	298	272	272.8	8.72	**7.72**	**7.72**	**7.72**	**7.72**
1-rc101	219	221	220.42	**−0.91**	0.00	0.00	0.00	0.00
1-rc102	266	256	255.84	3.76	**2.63**	**2.63**	**2.63**	**2.63**
1-rc103	266	250	242.81	**6.02**	**6.02**	**6.02**	**6.02**	**6.02**
1-rc104	301	275	274.6	**8.64**	**8.64**	**8.64**	**8.64**	**8.64**
1-rc105	244	227	225.25	6.97	7.79	7.79	**0.00**	7.79
1-rc106	252	247	227.93	**1.98**	3.17	3.17	3.17	3.17
1-rc107	277	256	255.69	**7.58**	**7.58**	**7.58**	**7.58**	**7.58**
1-rc108	298	283	248.22	**5.03**	6.71	6.71	**5.03**	6.71

**Table 22:** Results on Solomon benchmark with 2 days (*m* = 2 tours). Bold values denote the best results; negative values indicate the results exceeding the BK.

Instance	BK	KomoTrip profit		Gap (%)				
		Best	Average	KomoTrip (Proposed method)	ILS	ILS-SA	ILS-TS	ILS-SA-TS
2-c101	590	570	569.3	3.39	3.39	3.39	**1.69**	**1.69**
2-c102	650	641	607.98	**1.38**	1.54	3.08	1.54	1.54
2-c103	700	694	688.6	0.86	0.00	1.43	**−1.43**	0.00
2-c104	750	723	720.66	3.60	4.00	2.67	2.67	**1.33**
2-c105	640	635	618.8	**0.78**	1.56	3.13	1.56	3.13
2-c106	620	621	609.44	**−0.16**	0.00	0.00	0.00	0.00
2-c107	670	662	654.6	1.19	**0.00**	2.99	2.99	1.49
2-c108	670	668	655.27	0.30	**0.00**	**0.00**	**0.00**	**0.00**
2-c109	710	683	663.54	3.80	2.82	**0.00**	2.82	2.82
2-r101	330	306	294.81	7.27	6.67	6.67	**4.24**	6.67
2-r102	508	458	413.8	9.84	10.24	**8.86**	10.43	9.25
2-r103	513	499	472.8	2.73	3.70	2.92	**1.36**	3.12
2-r104	539	526	530.99	2.41	−0.19	−0.19	−0.19	**−0.37**
2-r105	430	414	383.1	3.72	3.26	3.72	**1.16**	3.26
2-r106	529	502	483.25	5.10	5.10	6.81	5.29	**4.35**
2-r107	529	501	464.98	5.29	5.29	8.51	6.99	**2.65**
2-r108	549	532	517.78	3.10	2.19	1.82	**1.09**	1.28
2-r109	498	488	481.79	2.01	1.81	1.81	**1.81**	**1.81**
2-r110	515	480	458.09	6.80	4.66	4.66	**3.30**	5.24
2-r111	535	497	476.79	7.10	7.10	**6.92**	**6.92**	8.04
2-r112	515	496	464.78	3.69	3.11	6.02	**1.94**	2.33
2-rc101	427	419	385.64	1.87	0.70	0.70	2.58	**0.70**
2-rc102	494	464	447.22	6.07	5.26	4.25	5.26	**3.44**
2-rc103	519	500	469.15	**3.66**	4.24	4.43	4.05	4.24
2-rc104	565	534	539.59	5.49	3.01	2.83	**2.12**	2.30
2-rc105	459	432	418.91	5.88	5.45	5.23	4.14	**3.49**
2-rc106	458	443	421.2	3.28	3.49	3.06	**0.44**	2.40
2-rc107	515	490	476.3	4.85	4.27	5.24	**2.33**	4.66
2-rc108	546	535	515.82	2.01	2.38	3.11	**1.47**	2.75

**Table 23:** Results on Solomon benchmark with 3 days (*m* = 3 tours). Bold values denote the best results; negative values indicate the results exceeding the BK.

Instance	BK	KomoTrip profit		Gap (%)				
		Best	Average	KomoTrip (Proposed method)	ILS	ILS-SA	ILS-TS	ILS-SA-TS
3-c101	790	803	769.56	**−1.65**	0.00	0.00	0.00	**1.27**
3-c102	890	863	854.63	3.03	3.37	**2.25**	**2.25**	3.37
3-c103	960	966	966.38	−0.63	**0.00**	**0.00**	−1.04	−1.04
3-c104	1,010	1,004	983.48	**0.59**	1.98	0.99	1.98	1.98
3-c105	840	834	816.46	**0.71**	1.19	1.19	1.19	1.19
3-c106	840	845	831.61	**−0.60**	1.19	1.19	0.00	1.19
3-c107	900	887	878.19	**1.44**	3.33	3.33	2.22	3.33
3-c108	900	885	867.28	**1.67**	3.33	3.33	3.33	2.22
3-c109	950	928	915.46	2.32	**2.11**	3.16	3.16	3.16
3-r101	481	458	455.71	4.78	5.41	2.91	**2.08**	5.41
3-r102	685	653	649.17	4.67	3.50	**2.63**	3.65	3.21
3-r103	720	716	684.56	0.56	**0.00**	1.53	0.14	1.53
3-r104	765	732	727.88	4.31	2.22	1.18	**0.39**	2.35
3-r105	609	563	531.34	7.55	6.24	9.03	6.57	**3.45**
3-r106	719	694	682.9	3.48	3.48	2.36	**0.97**	1.81
3-r107	747	703	680.05	5.89	4.28	5.49	**0.00**	5.35
3-r108	790	741	705.37	6.20	6.46	8.10	**5.06**	7.47
3-r109	699	652	624.4	6.72	5.87	8.30	**4.58**	8.01
3-r110	711	675	637.39	5.06	4.36	5.77	**3.66**	6.61
3-r111	764	760	722	**0.52**	0.92	1.57	1.18	1.57
3-r112	758	722	687.85	4.75	4.35	5.54	3.69	**3.56**
3-rc101	604	534	485.41	11.59	**9.44**	12.25	10.26	12.75
3-rc102	698	681	605.87	2.44	**2.29**	7.88	3.15	2.29
3-rc103	747	744	718.32	**0.40**	1.20	1.47	0.54	1.20
3-rc104	822	789	753.99	**4.01**	**4.01**	5.23	4.26	5.96
3-rc105	654	602	552.84	**7.95**	8.10	**7.95**	8.10	10.24
3-rc106	678	620	582.77	8.55	7.82	**6.19**	6.34	7.23
3-rc107	745	641	618.82	13.96	12.08	13.29	8.86	**4.97**
3-rc108	757	732	695.47	3.30	3.83	3.96	**0.66**	3.43

**Table 24:** Results on Solomon benchmark with 4 days (*m* = 4 tours). Bold values denote the best results; negative values indicate the results exceeding the BK.

Instance	BK	Komotrip profit		Gap (%)				
		Best	Average	KomoTrip (Proposed method)	ILS	ILS-SA	ILS-TS	ILS-SA-TS
4-c101	1,000	1,006	1,000.15	−0.60	1.00	1.00	0.00	**−1.00**
4-c102	1,090	1,118	1,076.98	**−2.57**	1.83	1.83	0.00	0.92
4-c103	1,150	1,173	1,166.37	**−2.00**	−0.87	−1.74	−1.74	−0.87
4-c104	1,220	1,230	1,214.8	**−0.82**	0.82	0.82	0.82	0.82
4-c105	1,030	1,034	1,028.52	−0.39	0.00	**−0.97**	0.00	**−0.97**
4-c106	1,040	1,049	1,045.7	**−0.87**	0.96	0.96	0.96	0.96
4-c107	1,100	1,089	1,056.25	**1.00**	2.73	4.55	3.64	3.64
4-c108	1,100	1,091	1,083.97	**0.82**	0.91	0.91	0.91	0.91
4-c109	1,180	1,127	1,144.28	4.49	**4.24**	**4.24**	**4.24**	**4.24**
4-r101	601	585	590.06	2.66	2.83	2.83	**2.16**	2.83
4-r102	807	795	759.98	**1.49**	3.97	3.97	2.97	4.09
4-r103	878	849	840.95	3.30	**0.91**	2.05	1.71	2.85
4-r104	941	911	864.74	**3.19**	5.10	5.84	4.04	5.21
4-r105	735	707	662.43	**3.81**	6.94	8.03	9.39	10.48
4-r106	870	867	841.67	**0.34**	2.07	0.80	1.03	1.72
4-r107	927	947	906.39	**−2.16**	0.43	0.22	1.08	1.08
4-r108	982	900	872.79	8.35	6.92	7.94	**6.82**	8.66
4-r109	866	816	771.81	5.77	**5.31**	**5.31**	6.81	7.74
4-r110	870	820	794.91	5.75	4.71	4.71	**1.72**	4.83
4-r111	935	888	845.65	5.03	4.71	5.45	**3.53**	5.99
4-r112	939	911	893.72	2.98	1.49	2.88	1.70	**0.43**
4-rc101	794	708	653.8	10.83	9.45	10.71	**8.94**	15.24
4-rc102	881	846	809.47	3.97	4.54	4.54	**3.97**	4.77
4-rc103	947	931	903.15	**1.69**	1.90	3.91	2.85	3.27
4-rc104	1,019	962	967.48	5.59	1.28	0.29	**−1.08**	0.69
4-rc105	841	805	772.6	4.28	3.80	3.80	**2.26**	3.57
4-rc106	874	840	780.62	3.89	3.78	**1.49**	5.03	6.06
4-rc107	951	924	894.45	2.84	2.21	1.58	0.63	**0.11**
4-rc108	998	909	832.34	**8.92**	10.02	9.92	12.22	11.82

Table 21 presents the comparative results for $m = 1$. KomoTrip secures 12 best solutions, demonstrating its competitiveness relative to the benchmark algorithms. Notably, KomoTrip is the only method that produces solutions surpassing the BK, for two instances, *i.e*., 1-c105 and 1-rc101, exceeding the previously reported benchmarks. In contrast, ILS-TS and ILS-SA-TS attain the largest number of best solutions, each with 17, highlighting their strength in consistently matching the BK. However, all benchmarking algorithms have no results that surpasses the BK.

Table 22 reports the gap results of the five algorithms for $m = 2$. KomoTrip achieves four best solutions, including one that surpasses the BK value. By contrast, ILS-TS demonstrates the strongest performance, attaining 15 best solutions. In addition to KomoTrip, the ILS-SA, ILS-TS, and ILS-SA-TS algorithms also generate solutions exceeding the BK. These findings suggest that while KomoTrip is not the leading method in terms of the number of best solutions, it remains competitive in producing the best solutions.

Table 23 presents the gap values for the Solomon instances with $m = 3$. KomoTrip demonstrates the strongest performance, attaining 10 best solutions, whereas ILS-TS get nine best solutions, while ILS-SA and ILS-SA-TS each yield four best solutions. Furthermore, KomoTrip exhibits competitiveness by generating three solutions that surpass the BK values, while ILS-TS and ILS-SA-TS each produce one such solution. Overall, KomoTrip consistently outperforms the four benchmark algorithms for the case of $m = 3$ tours.

Table 24 reports the gaps with respect to the BK for KomoTrip and four benchmark algorithms. For the case of $m = 4$ tours, KomoTrip achieves 13 best solutions, thereby outperforming all competing methods. Specifically, ILS yields three best solutions, ILS-SA attains 4, ILS-TS produces nine, and ILS-SA-TS generates five best solutions. Moreover, KomoTrip demonstrates a unique advantage by providing seven solutions that surpass the previously reported BK. Overall, the performance of KomoTrip improves with increasing values of $m$, as reflected in both the number of best solutions obtained and the number of instances surpassing the BK for the Solomon benchmark dataset.

For benchmarking on the Solomon dataset, we primarily report the profit values obtained, as direct comparison of running times across studies is confounded by differences in computing environments. To assess computational efficiency, we conducted additional experiments on six representative instances with $m = 3$ (3-c101, 3-c102, 3-r101, 3-r102, 3-rc101, 3-rc102), using ILS, ILS-SA, ILS-TS, and ILS-SA-TS as benchmarks. Each algorithm–instance pair was executed 50 times, and the average runtime values were recorded. The results, summarized in Table 25, indicate that KomoTrip consistently achieves faster running times than all benchmark algorithms.

**Table 25:** Running time comparison on selected Solomon instances with 3 days ( $m = 3$ tours). Bold values denote the best results.

Instance	KomoTrip (Proposed method)	ILS	ILS-SA	ILS-SA-TS	ILS-TS
c101	**0.7652**	2.4264	8.0309	14.6386	6.7806
c102	**0.7865**	2.5584	7.2947	16.5952	9.7651
r101	**0.5803**	2.2237	7.4741	14.6031	5.9822
r102	**0.6729**	2.2705	7.1642	14.9254	6.6234
rc101	**0.5679**	2.3013	6.9089	14.6431	6.1633
rc102	**0.6337**	2.4318	6.7427	14.2485	6.7158

Overall, KomoTrip demonstrated its ability to generate competitive profit values compared to other TOPTW algorithms, with improved performance for larger $m$ values. For $m = 1$ and $m = 2$, it still lags behind the other algorithms, but for $m = 3$ and $m = 4$, KomoTrip starts to produce competitive best solutions. Furthermore, in terms of running time, KomoTrip outperforms the benchmarking algorithms for this Solomon dataset. The high computational cost of ILS is mainly attributed to its sequential procedure and the exhaustive evaluation of neighboring candidate solutions. When combined with TS, the computational burden increases further, as the algorithm must process a wider neighborhood set to identify the best admissible (non-tabu) move. On the other hand, the integration of SA into ILS introduces additional overhead due to the acceptance criterion, while also expanding the search by evaluating a larger number of suboptimal solutions.

## Conclusion

This article introduces the KomoTrip method that has the ability to recommend optimal multi-day routes by accommodating weighted preferences of multi-attribute (criteria). To accommodate this requirement, we use the concept of Multi-Attribute Utility Theory (MAUT). The optimal solution search adopts the modified DKA for TOPTW. DKA is a modification of KMA (hereafter referred to as the original KMA) to solve discrete problems. In the optimization model, we define several constraint functions, penalty functions, and objective functions that accommodate weighted multi-attribute preferences. These objective functions reference the optimality of the solution (as a fitness function). We also introduce the split_itinerary algorithm (Algorithm 1) for per-day optimal route division, based on a greedy strategy.

The sensitivity analysis of the Komodo parameters $p$ and $smep$ confirms that the selected settings ( $p = 0.5$ and $smep = 5$) are consistent and robust. Across a wide range of tested values, the fitness values remain relatively stable (around 0.6), while running time shows only minor fluctuations. These findings indicate that KomoTrip’s performance is not overly sensitive to variations in $p$ and $smep$, and that the chosen parameters strike an effective balance between fitness achievement and computational efficiency. The sensitivity analysis also shows that KomoTrip’s running time remains relatively stable within certain parameter ranges (*e.g*., $p = 0.2 - 0.55$ and $smep = 10 - 20$), with only moderate fluctuations outside these intervals.

In experiments, we test the performance of the original KMA and KomoTrip. All experimental scenarios show that the original KMA is mostly superior in terms of fitness value compared to other algorithms. However, the main disadvantage of the original KMA lies in its worse running time compared to other algorithms. The superiority of original KMA in fitness value is due to the fact that the algorithm works in two adaptive phases and has three types of movements that optimize the solution search (HILE, MIME, LIHE). However, the adaptation of the original KMA to discrete problems requires a sorting process to be performed on each solution, which increases the time complexity of the algorithm. As a solution, KomoTrip is designed to offer a balance between the speed of running time and the quality of solutions that are relevant to the user’s preferences. In scenario 1 testing, KomoTrip consistently shows a significant advantage in terms of running time speed over comparable algorithms such as the original KMA, ACS, ACO, MOGA, DPSO, SA, TS, ILS, ILS-SA, ILS-TS, and ILS-SA-TS, although in terms of solution quality (fitness value), KomoTrip only consistently outperforms BSO, ILS, ILS-SA, ILS-TS, and ILS-SA-TS, but for the most part it is not significantly different from the other algorithms. In scenario 2 testing, similar results appear, where KomoTrip produces fitness values that are not significantly different from some of the other algorithms, but is dominant in running time speed. In this case, KomoTrip is a very competitive method for applications that require fast results. In scenario 3 testing, KomoTrip shows a stable fitness value pattern when the number of POIs reaches 20 or more, in line with the other algorithms (except BSO), but is consistently the fastest in terms of running time compared to ACO, ACS, MOGA, KMA, DPSO, SA, and TS.

Based on the evaluation results, although the original KMA outperforms all other algorithms in terms of fitness value, its weakness in running time makes it less ideal for the implementation of multi-day travel routes, where execution speed is a crucial factor. In contrast, KomoTrip is able to improve on this weakness by offering significant runtime efficiency, making it an effective solution for generating multi-day tour route recommendations that are relevant to users’ preferences. KomoTrip achieves competitive fitness values while showing superior runtime performance in Yogyakarta POIs dataset (proprietary dataset). Through advantages in execution speed and performance stability under various scenarios, this algorithm makes a significant contribution to recommending multi-day travel routes that incorporate weighted multi-attribute preferences.

Referring to the results of the benchmarking model on the Solomon dataset (public dataset for TOPTW), KomoTrip demonstrates its effectiveness in addressing the TOPTW by producing competitive profit values relative to established algorithms, with performance improving as the number of tours ( $m$) increases. Although its results remain less competitive for $m = 1$ and $m = 2$, KomoTrip attains stronger outcomes for $m = 3$ and $m = 4$, where it begins to generate a greater number of best solutions. Moreover, in terms of computational efficiency, KomoTrip consistently outperforms the benchmark algorithms across the Solomon dataset. Taken together, these findings highlight KomoTrip’s ability to balance solution quality with computational efficiency, making it a promising alternative for large-scale and time-constrained applications of the TOPTW.

Nevertheless, further development is needed to enhance the quality of solutions in terms of fitness value, in order to better compete with other strong algorithms. KomoTrip has practical potential for real-world deployment in tourism planning platforms (https://go-routes.com). It can serve as a backend engine for multi-day itinerary recommender systems, allowing users to customize their trips based on preferences, time windows, and daily constraints. In future research, KomoTrip could also be extended to integrate real-time data, such as traffic conditions, POI availability, or live user feedback, to enable more dynamic and adaptive route optimization for tourists and travel operators.

## Supplemental Information

10.7717/peerj-cs.3350/supp-1 Supplemental Information 1 Data POIs.

10.7717/peerj-cs.3350/supp-2 Supplemental Information 2 POI Schedule.

10.7717/peerj-cs.3350/supp-3 Supplemental Information 3 Time matrix POIs.

10.7717/peerj-cs.3350/supp-4 Supplemental Information 4 KomoTrip code.

## Additional Information and Declarations

### Data Availability

The following information was supplied regarding data availability:

The dataset is available at Zenodo: Baizal, Z. (2025). KomodoTrip POIs Dataset (Version 1) [Data set]. Zenodo. https://doi.org/10.5281/zenodo.17053243.

The program code is available at Zenodo: Baizal, Z. (2025). KomodoTrip Source Code (Version 1). Zenodo. https://doi.org/10.5281/zenodo.17053297.
