# Supplementary figures and images for: KomoTrip: a multi-day travel itinerary recommendation method based on the discrete komodo mlipir algorithm
Source: PeerJ Comput Sci. 2025 Nov 12;11:e3350. doi: 10.7717/peerj-cs.3350 (PMC12704617; doi:10.7717/peerj-cs.3350)

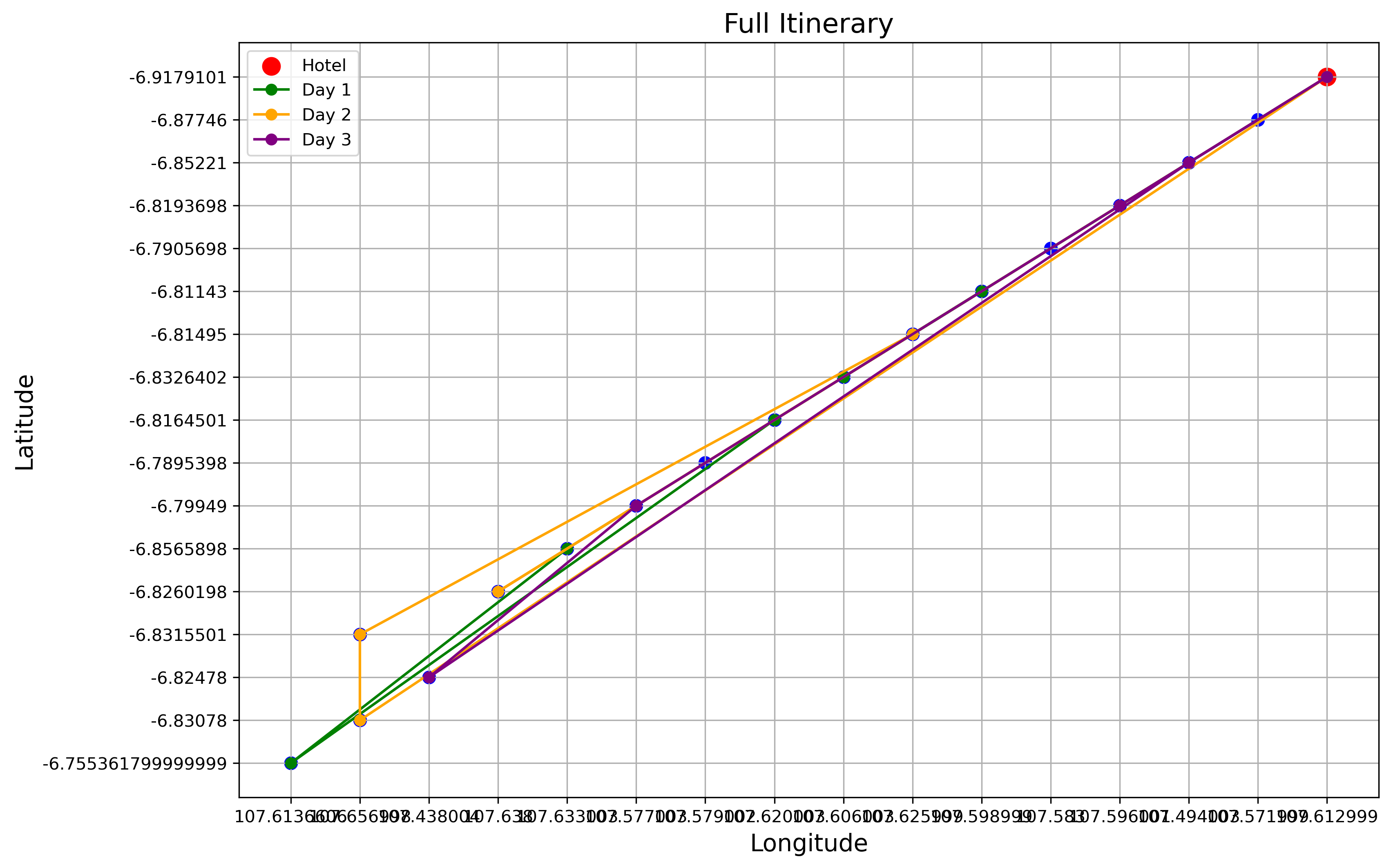

Supplement: Supplemental Information 4 [file peerj-cs-11-3350-s004.zip › travel-itinerary-recommendation-main/bandung_full.png]
